# The Raman fingerprint of cyclic conjugation: the case of the stabilization of cations and dications in cycloparaphenylenes†
†Electronic supplementary information (ESI) available: Experimental UV-Vis-NIR and Raman spectra of the radical cations and dications together with the complete characterization of optimized molecular geometries, charge distributions, computed spectra of all [*n*]CPPs discussed. See DOI: 10.1039/c6sc00765a


**DOI:** 10.1039/c6sc00765a

**Published:** 2016-04-06

**Authors:** Miriam Peña Alvarez, M. Carmen Ruiz Delgado, Mercedes Taravillo, Valentín G. Baonza, Juan T. López Navarrete, Paul Evans, Ramesh Jasti, Shigeru Yamago, Miklos Kertesz, Juan Casado

**Affiliations:** a Malta Consolider Team , Department of Physical Chemistry , Complutense University of Madrid , 28040 Madrid , Spain; b Department of Physical Chemistry , University of Málaga , 29071 Málaga , Spain . Email: casado@uma.es; c Department of Chemistry and Biochemistry and Materials Science Institute 1253 , University of Oregon , Eugene , Oregon 97403 , USA; d Institute for Chemical Research , Kyoto University , Uji 611-0011 , Japan; e Department of Chemistry and Institute of Soft Matter , Georgetown University , Washington , D.C. 20057-1227 , USA . Email: kertesz@georgetown.edu

## Abstract

Radical cations and dications of [*n*]CPP from *n* = 5 to *n* = 12 have been studied by Raman spectroscopy and density functional theory. Small [*n*]CPP dications owe their stability to the closed-shell electronic structure imposed by cyclic conjugation surpassing the destabilizing effect of ring strain and of the electron deficiency of the charged states. Large [*n*]CPP dications mitigate cyclic strain by forming diradicals. The Raman spectra reflect the balance among cyclic conjugation, cyclic strain and biradicaloid stabilization by finding an unexpected turning point in the frequency behavior of the G vibrational modes as a function of size. These Raman data represent the vibrational fingerprint of this rare form of conjugation which is central to stabilize both cations and dications of [*n*]CPP macrocycles.

## Abstract 2

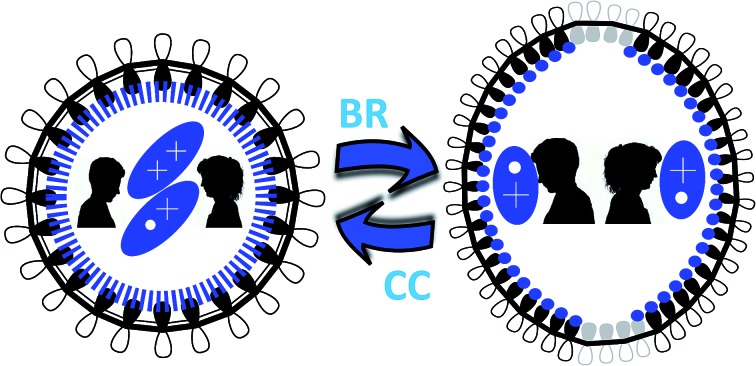
Cyclic conjugation and Biradical formation in charged cycloparaphenylenes are described by Raman spectroscopy and DFT.

## Introduction

The synthesis of cycloparaphenylenes ([*n*]CPPs, with *n* being the number of phenyl rings) was first attempted in 1934,^1^ but these were successfully synthesized for the first time by Jasti *et al.* in 2008.^2^ In only seven years every member of the [*n*]CPP series from [5]CPP to [18]CPP has been prepared.^2^–^4^ Nowadays the [*n*]CPP saga has become greatly diversified by the synthesis of many different types of functionalizations, however, “pure” [*n*]CPPs still represent the best molecular models to address important issues concerning the electronic structures of these exceptional cyclic π-conjugated molecules.^5^

**Scheme 1:**
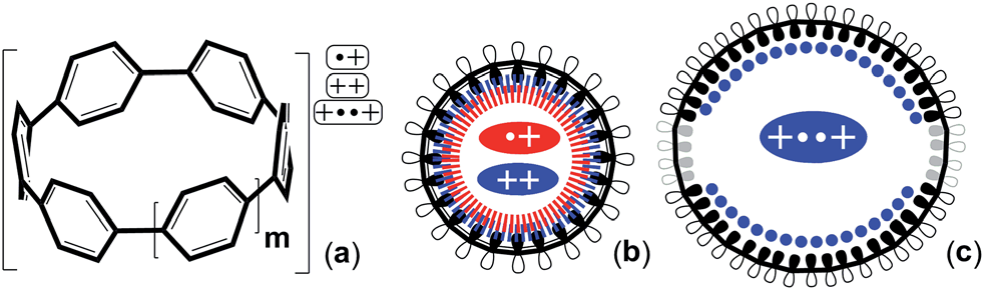
(a) Scheme outlining the studied charged species of [*n*]CPPs (*n* = *m* + 5, *m* = 0–7). (b) Top view of the 2p_*z*_ orbital orientation for cyclic conjugation in small charged [*n*]CPPs. (c) Top view of the symmetry breaking in large [*n*]CPP^2+^ after biradical formation. Red: radical cations, blue: dications.

The main distinctive features of [*n*]CPPs are the circular deformations of their π-electron structures and the cyclic strain imparted by the same curvature. In fact, in this [*n*]CPP series, [5]CPP accumulates an impressive cyclic strain energy of 119 kcal mol^–1^ (see Table S2† for computed strain) as the 360° curvature is shared only by five rings, yet neutral [5]CPP is fairly stable.^4^ A larger degree of destabilization is expected in their oxidized states (radical cations, [*n*]CPP˙^+^, and dications, [*n*]CPP^2+^) due to the removal of bonding electrons; yet, they are also quite stable as shown for [8]CPP˙^+^ and [8]CPP^2+^.^6^,^7^ Therefore, the fundamental question that arises is this: why are the smallest [*n*]CPP molecules in the oxidized states stable despite the increasing accumulation of ring strain compounded by the reduced number of available π-electrons (Scheme 1).

Recently, Yamago and Uchiyama addressed this issue for [8]CPP^2+^ by considering the [4*n*]annulene character of neutral [*n*]CPPs.^7^ Accordingly, they assumed that [8]CPP^2+^ is a 4(*n* – 1) + 2 π-electron system obtaining additional aromatic stabilization. The authors refer to such stabilization as in-plane aromaticity following the terminology based on the stability of [4*n*]annulene dications that have 4*n* – 2 = 4(*n* – 1) + 2 π-electrons.^8^ However, the stabilizing energy component provided by in-plane aromaticity in these [*n*]CPP^2+^ dications is expected to be small and therefore, *a priori*, it seems insufficient to justify the chemical robustness of the [*n*]CPP^2+^ series, especially for the highly strained smaller members, such as *n* = 5 and 6. Further in line with these energetic considerations, we also present evidence that [*n*]CPP monocations show intermediate behavior between the properties of neutral and dicationic [*n*]CPPs. Whether or not in-plane aromaticity is the main factor, the basic question is whether there is any direct or indirect experimental proof linked to the outstanding stabilization of smaller [*n*]CPP˙^+^/[*n*]CPP^2+^ cations that could be used to further decipher its nature and origin. Our conclusion is that we deal with a particular case of cyclic conjugation^9^ which is a rare form of thermodynamic stabilization and hence of interest in several fields of chemistry.

Yamago and Jasti^6^,^7^ have recently reported the properties of oxidized [8]CPP and other even members of the series mainly focusing on the electronic absorption spectra, ESR, VT-NMR and electronic structure calculations.^10^,^11^ Here, we analyze the complete, odd and even, cationic and dicationic series of [*n*]CPPs from *n* = 5 to 12. This work provides a unified perspective of the evolution of the electronic and molecular structures of these oxidized species based on Raman spectroscopy. This technique is highly valuable for the diagnosis of the π-conjugated and π-aromatic structures (as already described for neutral [*n*]CPPs)^12^,^13^ and enables us to characterize the specific factors responsible for the unique stability of the π-electronic structure of small [*n*]CPP˙^+^ cations and [*n*]CPP^2+^ dications. The approach relies on the size evolution of Raman data and structural (cyclic ring strain) and electronic (aromaticity) parameters.

## Results and discussion

The UV-Vis-NIR spectra of [8]CPP˙^+^ and [8]CPP^2+^ (Fig. S1–S3†) show that their Raman spectra are taken in near-resonance thus providing the unique vibrational fingerprint of each individual oxidized species. These experimental Raman spectra for [8]CPP˙^+^/[8]CPP^2+^ are compared in Fig. S2† with the (U)B3LYP/6-31G(d,p) predictions: the very good theory-experiment correlation supports the accuracy of the electronic structure calculations in terms of the energetics and the molecular structures as it will be discussed below. All [*n*]CPP˙^+^/[*n*]CPP^2+^ systems (*n* = 5–12) were thus characterized by UV-Vis-NIR electronic absorption spectroscopy and their bands assigned with TDDFT modelling.

In the Raman spectra of [8]CPP˙^+^/[8]CPP^2+^ the main bands at around 1600 cm^–1^ arise from CC vibrational modes describing an alternating stretching/shortening pattern of the consecutive CC bonds along the tangential direction of the macrocycle (G-type vibrations in carbon nanotubes) which are referred to here for the [*n*]CPPs as G-like modes.^12^ These have two G_A_1g__ and G_E_2g__ contributions, of which the G_A_1g__ one is a collective CC stretching vibration completely delocalized along the perimeter of the molecule (without vibrational nodes) and therefore highly sensitive to variations of the π-electron structure around the belt of the macrocycle (Fig. S5†). These G bands are our Raman probes for inquiring about molecular shapes and electronic structures in oxidized [*n*]CPPs.

**Fig. 1:**
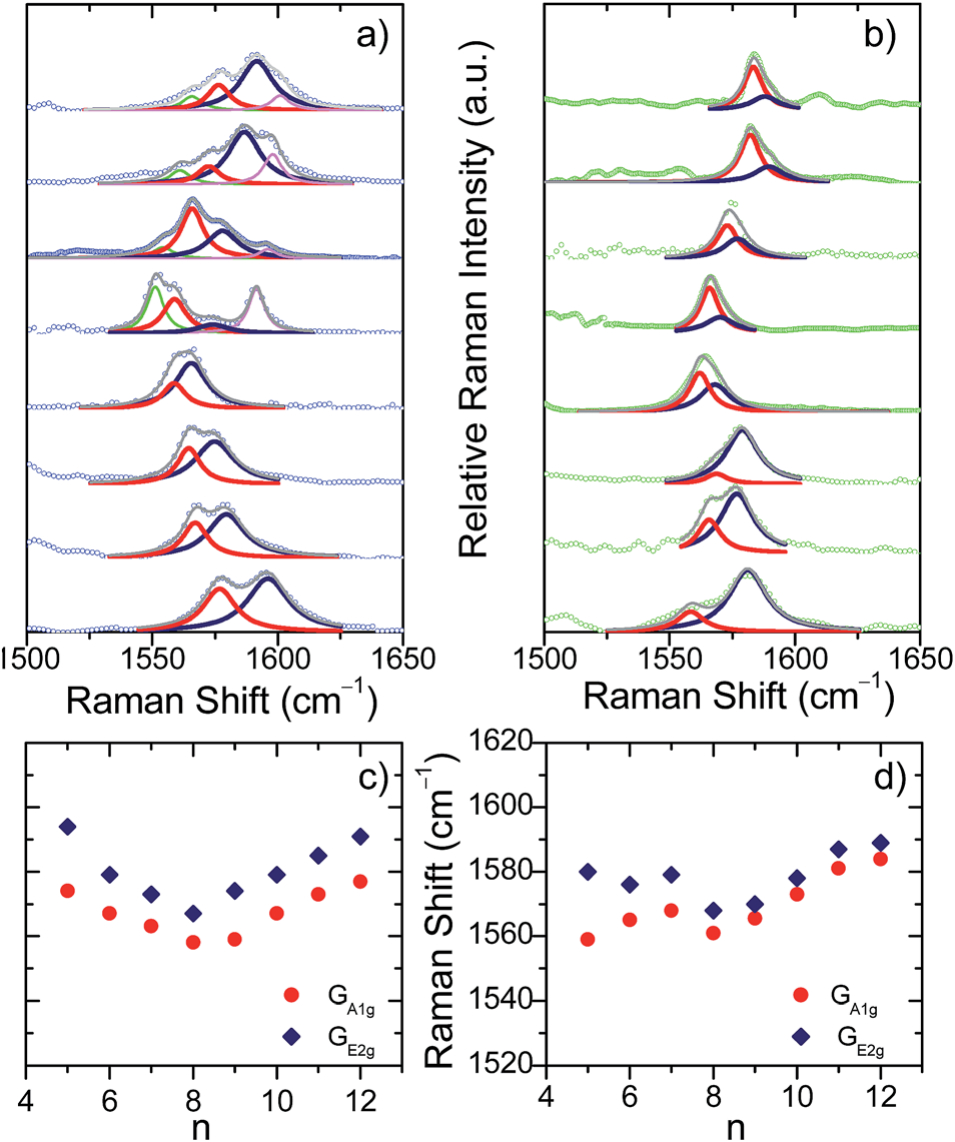
(a) G bands in the near-resonant Raman spectra in solution of [5]CPP^2+^ to [12]CPP^2+^ from the bottom to the top. Red and blue lines are the G_A_1g__ and G_E_2g__ Lorentzian deconvolutions, respectively. (b) The same for [*n*]CPP˙^+^. (c) and (d) G_A_1g__ and G_E_2g__ Raman shifts as a function of *n* showing a V-shape behaviour with [9]CPP at the vertex.

The evolution of the G bands in the Raman spectra of the neutral [*n*]CPPs (going from *n* = 12 to 6) has been already reported by us showing a continuous frequency downshift with decreasing *n*.^12^ This behavior has been ascribed to the continuous increase of quinonoidization on the benzenes as a result of their bending imposed by the strain in the macrocycle as its size decreases.^12^ In contrast to the neutral [*n*]CPPs, we present in Fig. 1 for the first time two distinctive and surprising trends on the G band frequencies for the dications: (i) the spectra for the large [*n*]CPP^2+^, [9]CPP^2+^ to [12]CPP^2+^, display several Raman bands (up to 4 in [12]CPP^2+^) whereas from [8]CPP^2+^ to [5]CPP^2+^ the spectra are simpler (two bands in [5]CPP^2+^); and (ii) the G_A_1g__/G_E_2g__ shifts follow a surprising V-shape behavior consisting of a progressive frequency downshift from [12]CPP^2+^ to [8]CPP^2+^ and from [5]CPP^2+^ to [8]CPP^2+^ with [9]CPP^2+^ being at the turning point.

Linear oligoparaphenylenes (LPPs) in their oxidized, cationic ([*n*]LPP˙^+^) and dicationic ([*n*]LPP^2+^) states are known to develop well-defined quinonoid structures along the π-conjugation path called polarons.^14^,^15^ These polarons have diagnostic Raman bands.^16^ Shorter [*n*]LPP^2+^ dications (*i.e.*, *n* < 4) have closed-shell singlet ground electronic states (S_0_), while larger ones with *n* > 4 start to stabilize an open-shell singlet structure driven by the re-aromatization of the innermost benzene rings giving way to a singlet diradicaloid pseudo-aromatic species.^16^ This behavior is reflected in the Raman spectra of [*n*]LPP^2+^ dications by the G band splitting shown in Fig. S11.† Concomitantly with the appearance of this diradicaloid character, the singlet–triplet gap (Δ*E*_ST_) is reduced and nearly vanishes for *n* > 8 owing to the large inter-radical separation. So far, no experimental proof of this closed-shell → open-shell transition in [*n*]LPP^2+^ dications has been seen in their Raman spectra given the limited number of available dicationic samples. However, this transformation of the quinonoid structure into a pseudo-aromatic singlet biradical has been demonstrated in a series of neutral quinonoid tetracyano oligo(*N*-annulated perylene) quinodimethanes.^17^

**Fig. 2:**
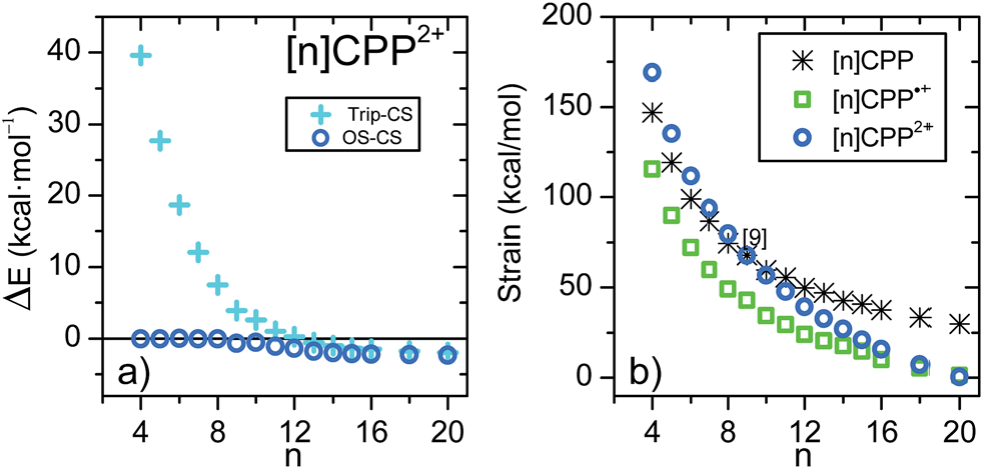
(a) DFT/(U)B3LYP/6-31G(d,p) relative energies (Δ*E*) between the singlet open-shell and singlet closed-shell (*E*_OS_ – *E*_CS_), and the triplet and closed-shell singlet (*E*_trip_ – *E*_CS_) of the [*n*]CPP^2+^ series. (b) (U)B3LYP/6-31G(d,p) strain energies calculated from the homodesmotic reaction in Scheme 2 and Fig. S11.† 18

Fig. 2 shows the variation of the energy difference between the closed-shell and open-shell electronic configurations of the [*n*]CPP^2+^ series. The behavior is qualitatively similar to [*n*]LPP^2+^ but quantitatively different. In Fig. 2a we observe that for *n* < 8, the closed-shell S_0_ state is by far the most stable configuration with a Δ*E*_ST_ gap to the first triplet excited state of ≈20 kcal mol^–1^. For larger [*n*]CPP^2+^, *n* > 9, the open-shell biradical form becomes the S_0_ ground electronic state with a small but non-vanishing Δ*E*_ST_ gaps revealing how the cyclic structure, in contrast to [*n*]LPP^2+^, avoids terminal spin accumulation. These data support the biradical character of the ground state as suggested by Yamago *et al.* for [10]CPP^2+^ and [12]CPP^2+^ based on H-NMR.^10^

**Scheme 2:**
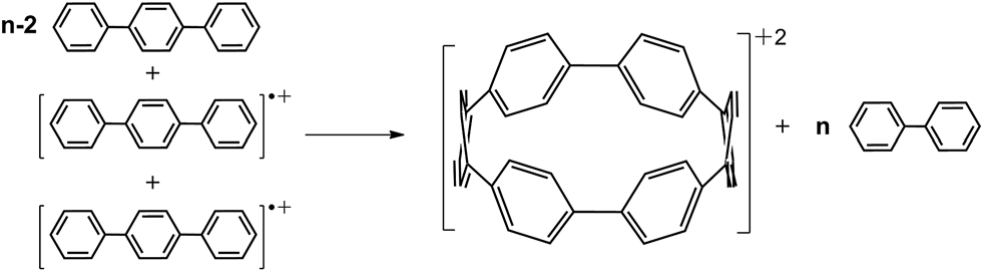
Homodesmotic reaction for the calculation of strain energies of [*n*]CPP^2+^ dications.

In Fig. 2b we present the calculated macrocyclic strains of the [*n*]CPP^2+^ dications which have been calculated considering the corresponding homodesmotic reaction, as shown in Scheme 2 and Fig. S11† adapted from a similar procedure for the neutral [*n*]CPPs.^18^ In our case for the dications, we consider two radical cations in the reactants which thus include the +2 ionic state for the final dication. Interestingly, while the *n* < 9 [*n*]CPP^2+^ dications have larger strain energies, by up to +20 kcal mol^–1^ than the neutrals, for *n* ≥ 9, the biradical [*n*]CPP^2+^ dications have less strained structures compared to the neutral molecules. This difference can be explained in terms of the bending of the six-member rings: in the neutrals, cyclic strain energy is proportional to the reduction in aromaticity of the benzene units. For the dications, oxidation itself causes a partial reduction of the aromaticity of the benzene rings and consequently the energy cost of the local deformation is smaller. For *n* < 9, the reason for the energy strain being larger in the dications is associated with the repulsive electrostatic term which is not present in the neutrals. These repulsive interactions are larger in the smaller molecules where the two positive charges are more confined. Nonetheless, this additional electrostatic term in these small dications provides a challenge for understanding their unexpected stability. Comparing the size dependent behavior of strain and biradical formation in Fig. 2b, we deduce that in larger [*n*]CPP^2+^ dications the formation of the diradicaloid configuration takes place concomitant with the mitigation of the ring strain. Both the stability switch over in the strain values and the sudden appearance of diradical character occur at the same critical *n* = 9 size which is also the turning point for the frequency behavior for the structurally sensitive Raman G bands.

**Fig. 3:**
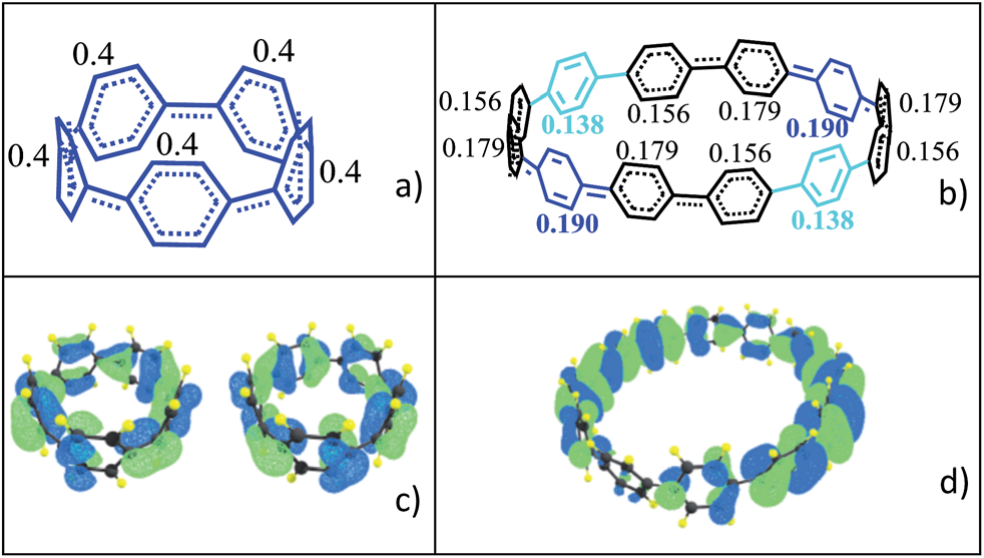
DFT-(U)B3LYP/6-31G(d,p) data: per ring charge distribution and valence bond structures from optimized molecular geometries for (a) [5]CPP^2+^ and (b) [12]CPP^2+^. (c) Degenerate HOMO and HOMO–1 orbital topologies for [5]CPP^2+^, and (d) HOMO of [12]CPP^2+^.

The transition as a function of size of the macrocycle from small and highly strained (singlet closed-shell) to large and less strained (open-shell) has a significant impact on the molecular geometries as represented in Fig. 3 for the two extreme cases of [5]CPP^2+^ and [12]CPP^2+^. In the smaller [*n*]CPP^2+^ dications all six member rings are nearly equivalent, whereas from [9]CPP^2+^ on the macrocycles show domains with different aromatic-quinonoid characters in the CC bond length alternation (BLA) pattern resulting in an overall symmetry loss of the circular shape towards an oval structure with increasing of *n* (see Table S3 and Fig. S13† for the geometries of the whole series). Similar symmetry breaking has been observed for dicationic cyclic oligothiophenes.^19^ The relevant frontier molecular orbitals shown in Fig. 3 further reveal the transition between small and large [*n*]CPP^2+^ dications: the HOMO/HOMO–1 degeneracy which is present for *n* < 9 is broken for *n* ≥ 9 (Table S4†).

**Fig. 4:**
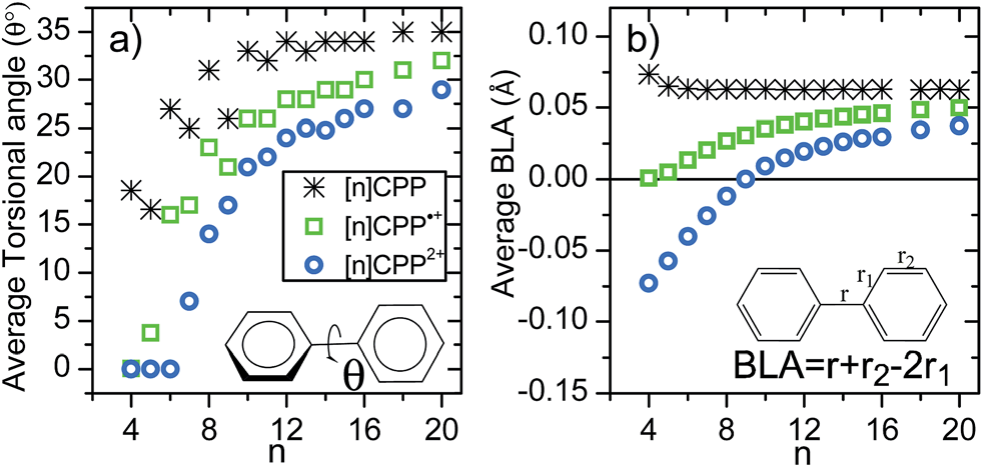
(a) Size evolution of the average interring torsional angles (*θ*, see inset). (b) Size evolution of the average BLA parameter (see inset for the definition of BLA).

Another relevant structural feature of the [*n*]CPPs is the distribution of conformations between neighbouring benzene rings provoked by the hydrogen–hydrogen steric repulsions forcing phenyl to phenyl torsions (*θ*) in the neutral molecules. These produce an alternating pattern of torsions for even *n* values.^13^ Compared to the neutral series, the situation in the [*n*]CPP^2+^ series is substantially different. The evolution of the average torsion in Fig. 4a displays a significant change by oxidation, decreasing from neutrals to cations and even more from cations to dications. Thus, the smaller dications ([4]CPP^2+^–[6]CPP^2+^) have close to zero *θ* values that increase up to 14° in [8]CPP^2+^. This is important since smaller torsions enhance π-conjugation between neighboring benzenes consistent with a larger double bond character for the interring bond, *r*. Fig. 4b shows negative BLA values indicative of a significant quinonoid character for *n* < 9 in the dication series. It is this decrease of the BLA that shows up in the Raman shifts providing the experimental evidence.

The unusual experimental Raman spectral behavior described in Fig. 1 is nicely explained by taking into account all these structural effects: (i) the existence of an increased number of Raman bands in larger [*n*]CPP^2+^ dications is the result of the appearance of aromatic and quinonoid domains lowering the molecular symmetry leading to new Raman-active bands. (ii) The overall [12]CPP^2+^ → [9]CPP^2+^ frequency downshift corresponds to the gradually increasing contributions of the local quinonoid structure of the benzene rings with decreasing *n*, similar to the structural and Raman spectroscopic behavior described for the neutral [*n*]CPP series.^12^,^13^ For instance, in [12]CPP^2+^ the strain and bending effects are small and the G frequency therefore is the highest among *n* = 9–12 members of the series. (iii) When moving from [8]CPP^2+^ to [5]CPP^2+^ the closed-shell structure is stabilized (the open shell diradical character essentially disappears leading to the increase in absolute value of the BLA as seen in Fig. 4b) provoking the collective circumferential C=C/C–C vibration G mode frequencies to upshift as of *n* decreases from *n* = 9 to *n* = 5.

As already mentioned before, in the π-electronic structures of [*n*]CPP, one can identify substructures of the *cis*–*trans* [4*n*]annulene type^7^ (*i.e.*, considering four π-electrons per benzene, 4*n* in total) and from this point of view the [*n*]CPP^2+^ annulene-like dications feature 4*n* – 2 = 4(*n* – 1) + 2 Hückel π-electrons in a conjugated circuit. This electron counting has been referred to as in-plane aromaticity, a concept also known as superaromaticity.^20^ Under this hypothesis, we observe that the triplet states of [*n*]CPP^2+^ dications represent electronic configurations with 4(*n* – 1) bonding π-electrons so the Δ*E*_ST_ gap of >20 kcal mol^–1^ for [5]CPP^2+^ in Fig. 2a emerges as a result of the energy required to break its closed-shell electronic structure. As the macrocycle size decreases its Δ*E*_ST_ gap increases indicating the effective strength of the bonding within the superaromatic conjugated π-electrons.

The radical cations, [*n*]CPP˙^+^, have been also characterized by UV-Vis-NIR (Fig. S4†) and Fig. 1 also display their resonant or near-resonant Raman spectra where, similarly to the dications, a close to a V-shape behavior in the G frequency bands as a function of *n* is found. The turning point is also between [8]CPP˙^+^ and [9]CPP˙^+^. From theoretical calculations in Fig. 4 we see that for the larger [*n*]CPP˙^+^ cations (*n* > 8), their average dihedral interring torsion angles are larger than in the smaller analogues meaning that an increasing tendency to localize the charge among the benzene rings is expected which would cause the Raman bands to upshift going from [9]CPP˙^+^ → [12]CPP˙^+^. This trend is to be contrasted with the *n* < 9 cases where the frequency upshifts of the radical cations originate from the cyclic delocalization of the charge over the whole molecular cylinder (Table S5†). This full delocalization, such as in the dications, reinforces the C=C/C–C bond alternation path (see BLA evolution in Fig. 4b) and leads again to the Raman upshift going from [8]CPP˙^+^ to [5]CPP˙^+^. In regard to strain energies, the behavior [*n*]CPP˙^+^ cations (4*n* – 1 π-electrons) is intermediate between the neutrals (4*n* π-electrons) and the dications (4(*n* – 1) + 2 π-electrons) showing some aspects of both.

Finally, it is interesting to jointly analyze in Fig. 4b the evolution of the bond length alternation as a function of *n* for neutral, cationic and dicationic [*n*]CPPs. The BLA of the neutrals is positive and barely changing with *n* indicating an overall aromatic character of the benzene units. The BLA in small [*n*]CPP˙^+^ cations reaches a value showing an intermediate structure. The dications display more pronounced changes in the BLA. For *n* > 9 the BLA is positive indicating an overall pseudo-aromatic structure in line with the open-shell biradical state. For [9]CPP^2+^the BLA close to zero representing the turning point towards a negative BLA value. For smaller *n* < 9 the BLA attains larger negative values indicating an increased double bond character for the interring bond (*r*) due to electron pair localization in the closed-shell configuration. Comparing the absolute values, an increasingly bond length alternating π-conjugated structure is in consonance with the upshift of the Raman frequencies of the dication from [9]CPP^2+^to [5]CPP^2+^. These results are complemented by the calculations of the Nucleus-Independent Chemical Shifts (NICS) for each [*n*]CPP at the cavity center, NICS(C) (shown in Fig. S16†). There is a rapid decrease of NICS(C) with the incremental addition of the positive charge (neutral, radical cation and dication) and with decreasing *n* reaching NICS(C) values of –20 ppm for [5]CPP^2+^, from which it can be inferred that a strong ring current is being induced in the cavity due to the efficiency of the cyclic conjugation.

Taking the data in the cation and dication series together, a similar V-shape Raman frequency behavior is found for both. However, while [*n*]CPP^2+^dications correspond to the 4(*n* – 1) + 2 Hückel aromatic formula, [*n*]CPP˙^+^ cations are formally non-aromatic, so the stabilizing effect that compensates the macrocyclic strain in the [*n*]CPP˙^+^ cations cannot be aromatic stabilization alone but it must be partly the result of charge delocalization over the macrocycle. In-plane conjugation as used by other authors^7^ in the literature seems to be an ambiguous term because a plane of conjugation does not exist for [*n*]CPPs (the effect spreads out over the whole nearly cylindrical molecule). Therefore, the stabilizing charge delocalization effect occurring in cations and dications here, we think, is a manifestation of C=C/C–C conjugation in a cyclic geometry, or cyclic conjugation.^8^

## Experimental

### Oxidation titrations

Et_3_O^+^SbCl_6_^–^ was used to generate the cations and dications in 10^–4^ M solution of [*n*]CPPs in CH_2_Cl_2_. Oxidations were monitored by UV-Vis-NIR absorption using a Cary 5000 spectrophotometer.

### Raman spectroscopy

Raman measurements at room conditions of [*n*]CPP˙^+^ and [*n*]CPP^2+^ were done at resonant and near resonant conditions with 532 nm, 785 nm or 1064 nm laser excitations, using an Invia Reflex Raman RENISHAW and a Ram II Bruker FT-Raman spectrometers. The bands have been fitted with Lorentzian curves (FWHM = 11–13 cm^–1^ for the G_A_1g__ band and 14–20 cm^–1^ for the G_E_2g__ band).

### Theoretical calculations

Density Functional Theory (DFT) quantum-chemical calculations were performed as implemented in Gaussian 09.^22^ Energy values and geometrical parameters refer to the geometry-optimized structures using the B3LYP/6-31G(d,p) method, the minima were checked by frequency calculations. The unrestricted (U)B3LYP/6-31(d,p) approach was used for open shell cations and dications. For open shell ground-state of the dications, broken symmetry spin-unrestricted theory with the guess = mix keyword was used. The theoretical Raman activities refer to non-resonant conditions, while the experiments were done on or near resonance. In addition, the broken-symmetry DFT approach has problems with the prediction of Raman spectra for open-shell diradicals and the experimental *versus* theoretical spectra do not agree well for *n* > 9 (see the ESI file for further details†). TDDFT computations were done for the UV-Vis absorption spectra using TD-(U)B3LYP/6-31G(d,p), and NICS calculations were done at the (U)B3LYP/6-311++G(2df,p) GIAO level.

## Conclusions

In summary, the complete series of cations and dications of [*n*]CPPs from *n* = 5 to *n* = 12 have been studied. Small [*n*]CPP^2+^dications owe their stability to the closed-shell structure imposed by the efficient cyclic conjugation which softens the impressive increase in ring strain. In this regard, these small [*n*]CPP^2+^dications are real models for investigating cyclic conjugation instead of relying on the purely theoretically constructed cyclacenes.^21^ As the size increases, cyclic conjugation vanishes and large [*n*]CPP^2+^ dications mitigate cyclic strain by forming biradicaloid structures. The balance between cyclic conjugation, cyclic strain and biradicaloid transformation is evidenced in the Raman spectra by the detection of a surprising V-shape behavior of the G band frequencies.

## Supplementary Material

Supplementary information
