# Supplementary material for: The Raman fingerprint of cyclic conjugation: the case of the stabilization of cations and dications in cycloparaphenylenes
Source: Chem Sci. 2016 Apr 6;7(6):3494–9. doi: 10.1039/c6sc00765a (PMC6007183; doi:10.1039/c6sc00765a)
Supplement: Supplementary file 1 [file SC-007-C6SC00765A-s001.pdf]

Electronic Supplementary Material (ESI) for Chemical Science.
This journal is © The Royal Society of Chemistry 2016

Supporting information

The Raman Fingerprint of Cyclic Conjugation: The case of the Stabilization of Cations and Dications in Cycloparaphenylenes

Miriam Peña Alvarez,[a] M. Carmen Ruiz Delgado,[b] Mercedes Taravillo,[a] Valentín G. Baonza,[a] Juan T. López Navarrete,[b] Paul Evans,[c] Ramesh Jasti,[c] Shigeru Yamago,[d] Miklos Kertesz,*[e] Juan Casado*[b]

a MALTA-Consolider Team, Department of Physical Chemistry, Chemistry Faculty, University Complutense of Madrid, 28040 Madrid, Spain.

b Department of Physical Chemistry, University of Málaga, Campus de Teatinos s/n, Málaga, 29071, Spain

c Department of Chemistry and Biochemistry and Materials Science Institute 1253, University of Oregon, Eugene, Oregon 97403, USA.

d Institute for Chemical Research, Kyoto University, Uji 611-0011, Japan.

e Department of Chemistry and Institute of Soft Matter, Georgetown University, 37th and O Streets, NW, Washington, D.C. 20057-1227, USA.

Experimental and theoretical details

Preparation of the oxidized species. [n]CPPs with *n* = 7, 8, 9, 10, 11 and 12 were synthesized by using a synthetic strategy through multinuclear arylplatinum complexes,[1] while [5]CPP and [6]CPP were synthesized through Suzuki–Miyaural cross-coupling/macrocyclization.[2,3] Triethyloxoniumhexa-chloroantimoniate ($Et_3O^+SbCl_6^-$) was used to generate the cations and dications in a free oxygen and water atmosphere at room temperature. All the reactions were done in solution of [n]CPP, 10^{-4} M, using dichloromethane as solvent, CH_2Cl_2, by stepwise addition of the salt $Et_3O^+SbCl_6^-$ (10^{-4} M). Chemical oxidations were monitored by in situ UV-Vis-NIR electronic absorption spectroscopy using a Cary 5000 UV-vis-NIR spectrometer.

Raman spectroscopy. Raman measurements at room temperature of the different [n]CPPs cations and dications were conducted under near-resonant or resonant conditions by selecting the excitation wavelengths that mostly fulfill one of the electronic absorption bands of the UV-Vis-NIR spectra. Available Raman excitation wavelengths are at 532, 633, 785 nm of an Invia Reflex Raman RENISHAW microscope. The 1064 nm excitation FT-Raman spectra were obtained with a Ram II spectrometer from Bruker. The experimental bands have been fitted with Lorentzian profiles with FWHM ranging between 12 and 15 cm^{-1}.

Summary of the excitation wavelengths used for the different Raman measurements carried out for neutral, cations and dications of the [n]CPPs.

n	**[n]CPP**	**[n]CPP$^{\bullet+}$**	**[n]CPP^{2+}**
5	785 nm	1064 nm	1064 nm
6	785 nm	1064 nm	1064 nm
7	785 nm	1064 nm	532 nm
8	785 nm	532 nm	1064 nm
9	785 nm	532 nm	1064 nm
10	785 nm	532 nm	1064 nm
11	785 nm	532 nm	1064 nm
12	785 nm	532 nm	1064 nm

Theoretical calculations. Quantum-chemical calculations were performed with Density Functional Theory (DFT) as implemented in the Gaussian 09 package.[4] All reported energy values and geometrical parameters refer to the geometry-optimized structures using the B3LYP/6-31G(d,p) method. All minima have been confirmed by vibrational calculations with all frequencies being real. The unrestricted UB3LYP/6-31(d,p) approach was used for cations and dications. The ground state configuration for all neutral [n]CPPs and for dications with $n<9$ are closed shell solutions. The geometries for the open-shell singlet dications were obtained from previous optimization of the triplet dications. For the open-shell singlet electronic ground state structures of the dications, we used the broken symmetry method with the keyword "guess = mix" at the (U)B3LYP level. Theoretical Raman spectra were represented with a FMHW of 3 cm^{-1}. In order to consider the discrepancy between theory and experiments (especially for n>9), two main reasons should be taken into account: i) while the experiments are taken in resonance or near-resonance conditions, the computed Raman activities refer to non-resonant conditions. This deficiency is reflected in the relatively poor agreement with experiment, especially for the theoretical Raman spectra for n>8 (See Figures S6 and S7). Ii) The broken symmetry DFT approach for the prediction of Raman spectra fails for open-shell diradicals, so in our case for n>8 the theoretical/experimental comparison is weak while for closed-shell systems (n<9) the comprsion is quite good. . In despite of this, we wish to be consistent and show these theoretical spectra from n>9.

Nucleus-Independent Chemical Shifts (NICS) calculations were done at the (U)B3LYP/6-3112+G(2df,p) level. All theoretical frequencies were scaled by a uniform factor of 0.96. All NICS values were computed at the (U)B3LYP/6-311+G(2df,p) level using the gauge including atomic orbitals (GIAO).

Chemical oxidation of [8]CPP

Figure S1 shows the UV-Vis-NIR spectra for [8]CPP, [8]CPP$^{\bullet+}$,[8]CPP^{2+}, and [8]CPP$^{\bullet3+}$ (obtained as described in the previous section). Vertical excitations in Figure S2 were theoretically calculated with the TD-DFT theory using 40 states. In general, the agreement between experimental and theoretical data is very good.

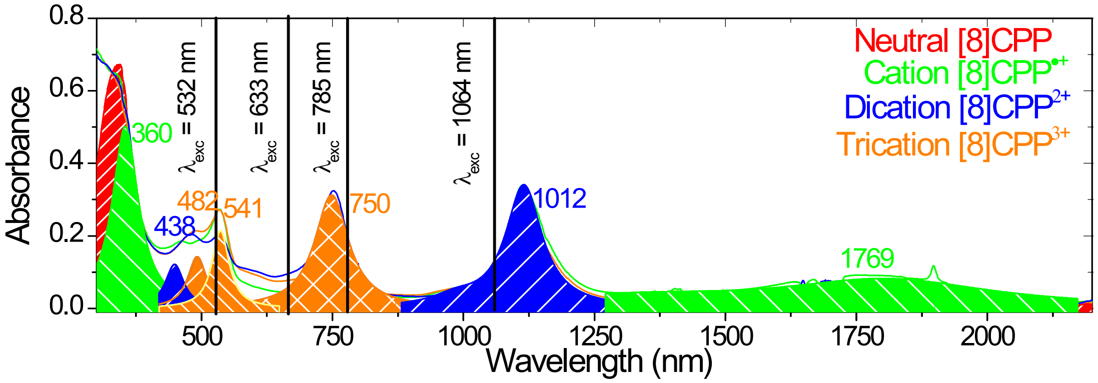

Figure S1. In situ UV-Vis-NIR electronic absorption spectra of [8]CPP obtained by chemical oxidation. Neutral [8]CPP is in red, radical cation [8]CPP$^{\bullet+}$ in green, dication [8]CPP^{2+} in blue and trication [8]CPP^{3+} in orange. The λ_{exc} values marked by vertical lines denote the laser excitation wavelengths used to obtain the resonant (or near-resonant) Raman spectrum for each species.

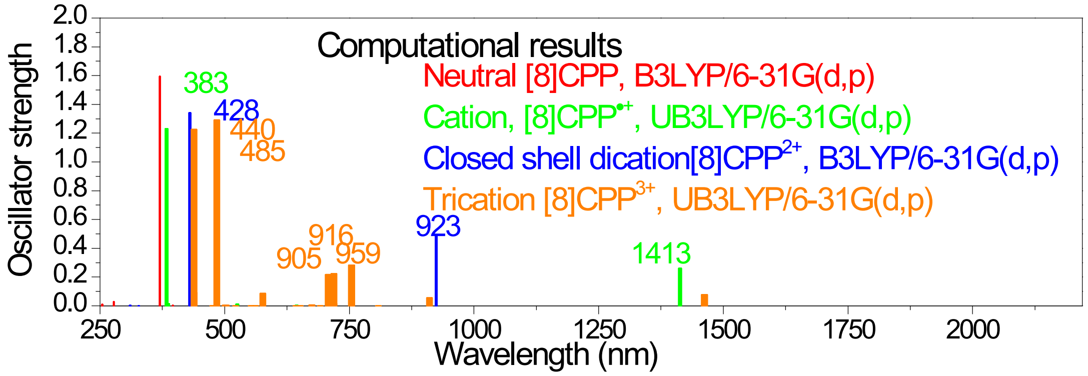

Figure S2. TD-(U)B3LYP/6-31G(d,p) vertical excitations for the different oxidation states of [8]CPP obtained at the optimized (U)B3LYP/6-31G(d,p) geometries using 40 excited states.

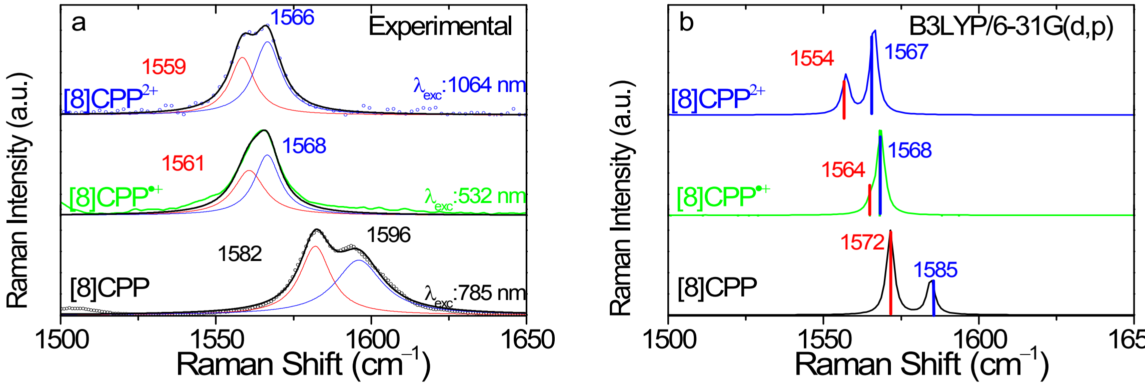

*Figure S3***.** Raman spectra of neutral [8]CPP and its radical cation and dication in the 1500 - 1650 cm^{-1} spectral region. a) Experimental Raman spectra. b) (U)B3LYP/6-31G(d,p) computed scaled spectra at the optimized geometry of oxidized species. There are two major contributions in this region: the G_{A1g} low frequency feature is in red, and the G_{E2g}, high frequency feature is in blue.

UV-Vis-NIR characterization of oxidized [n]CPP

The cationic and dicationic species of [n]CPP from n=5 to 12 are characterized by UV-Vis-NIR absorption spectroscopy. In Figure S4 we show a comparison of these experimental spectra with the theoretical excitation energies predicted by TD-(U)B3LYP/6-31G(d,p) for all these species. Radical cations show two main bands, one broad band in the NIR region and another in the UV region. The NIR band is due to the mixture of the HOMO → SOMO and the HOMO-1 → SOMO transitions. These absorptions shift to longer wavelengths as n increases. This behavior is well predicted by theory. The smaller [n]CPP dications have two bands that appear between those of the radical cations. For dications larger than [9]CPP^{2+} the two bands of the dications start to progressively resemble those of the radical cations although the dicationic bands stay between those of the cationic species. Theoretical spectra also reproduce this behavior. For the dications, the band at longer wavelength in the NIR originates from the HOMO –LUMO transition. The second band is in the UV-Vis and it corresponds to the HOMO-1→LUMO+1 transition.

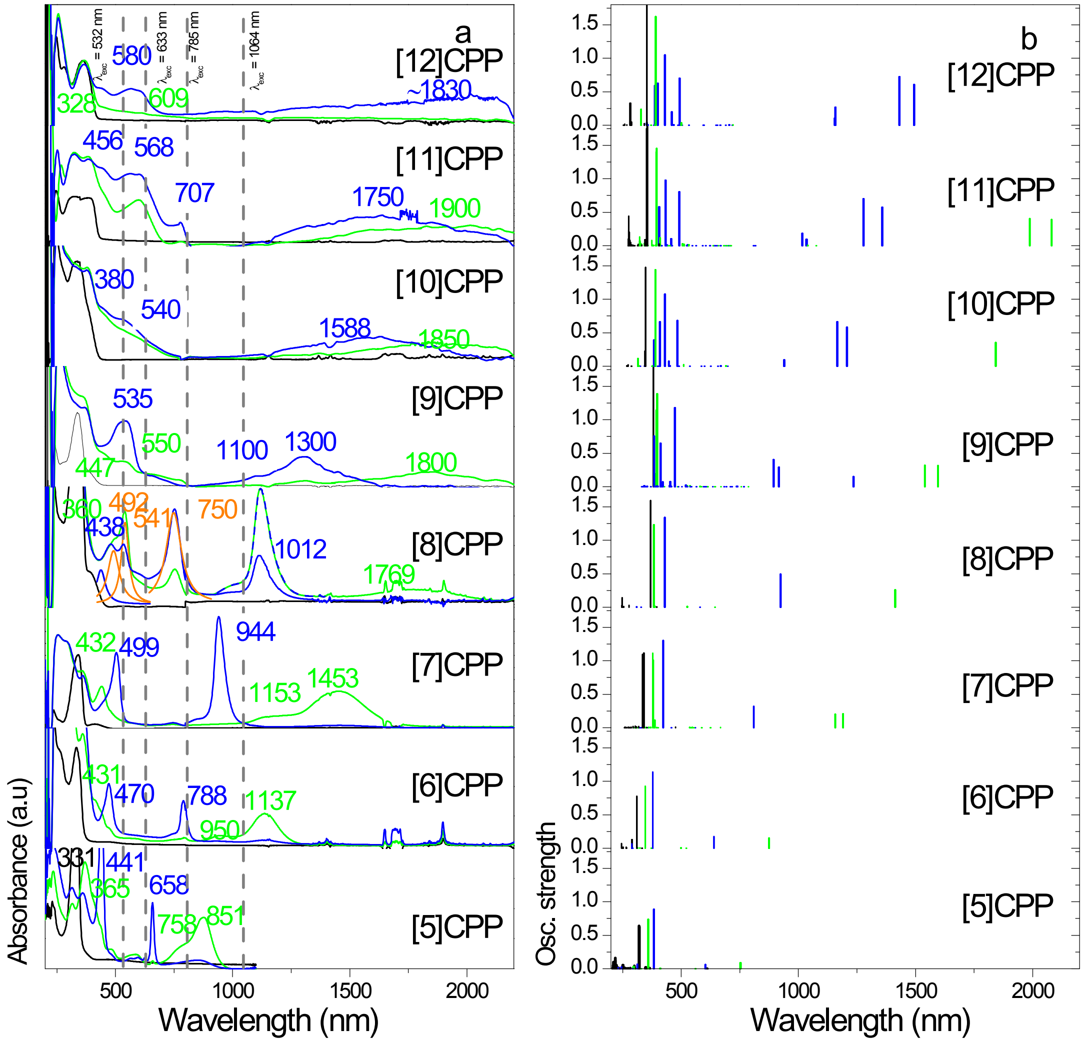

Figure S4. Electronic absorption spectra for neutral, cationic and dicationic species of [n]CPPs (from n=5 to n=12). (a) Experimental, and (b) computed vertical excitations at the TD-(U)B3LYP/6-31G(d,p) level. Red refers to the neutral, green to the radical cationic and blue to the dicationic species. The orange spectrum of [8]CPP corresponds to its radical trication, [8]CPP^{3+}.

Raman spectra of [n]CPP^{2+} dications

This section is devoted to the analysis of the Raman spectra of the dications of [n]CPPs. In Figure S5 we present the eigenvectors of the G modes for the representative cationic and dicationic species of [8]CPP. We compare the experimental and computed Raman spectra in the 1100-1700 cm^{-1} spectral region for the dications in Figure S6. The Raman shifts of the respective G bands are summarized in Figure S7.

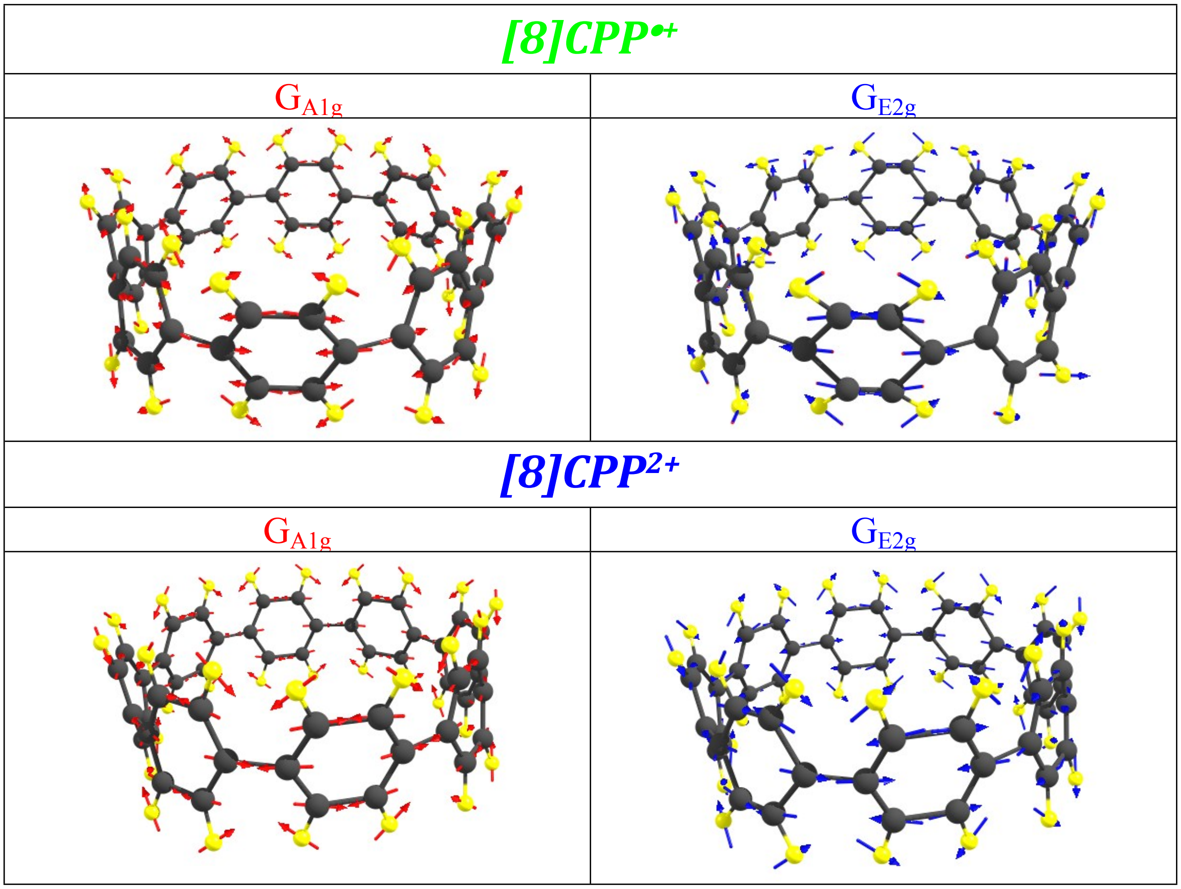

Figure S5. Vibrational eigenvectors from the theoretical (U)B3LYP/6-31G(d,p) Raman spectra of the G_{A1g} (the strongest) and G_{E2g} (weaker) bands of [8]$CPP^{\bullet+}$ (top) and [8]CPP^{2+} (bottom).

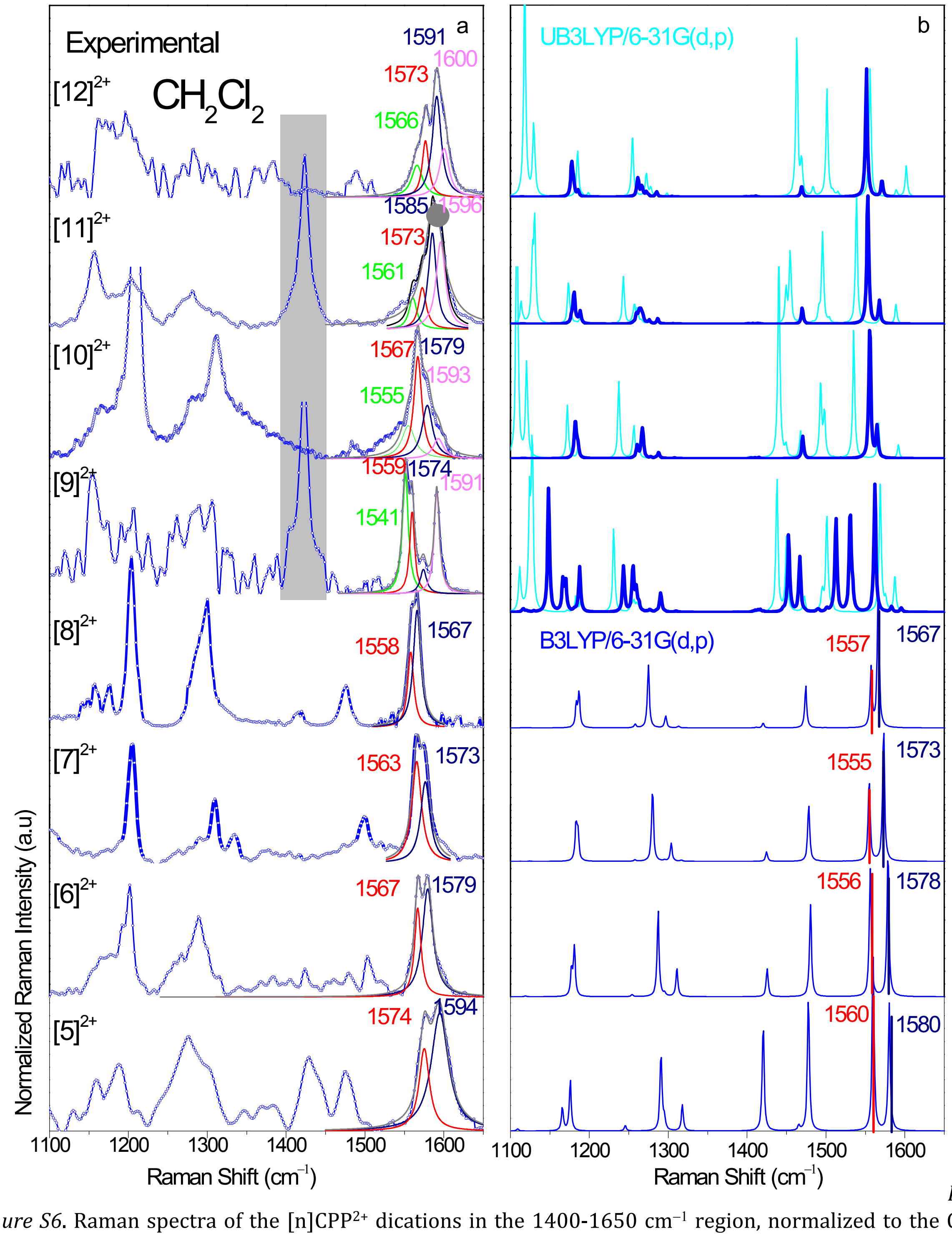

Figure S6. Raman spectra of the [n]CPP^{2+} dications in the 1400-1650 cm^{-1} region, normalized to the G_{A1g} band. a) Experimental spectra. Shadowed grey region corresponds to the most intense Raman band of the CH_2Cl_2 solvent which is detected in near-resonant spectra or when full resonance is not fulfilled. The G_{A1g} band is in red, the G_{E2g} one is in dark blue. Green and pink bands correspond to those which appear in the open shell singlets, and these are referred to as G_{OS1} and G_{OS2}, respectively. b) Computed spectra. Dark blue is obtained with B3LYP/6-31G(d,p). The light blue computed spectra (for *n*>9) refer to UB3LYP/6-31G(d,p) computation which do not reproduce the spectra well. The frequency predictions for n>8 are por and are not shown here. See also Fig. S7."

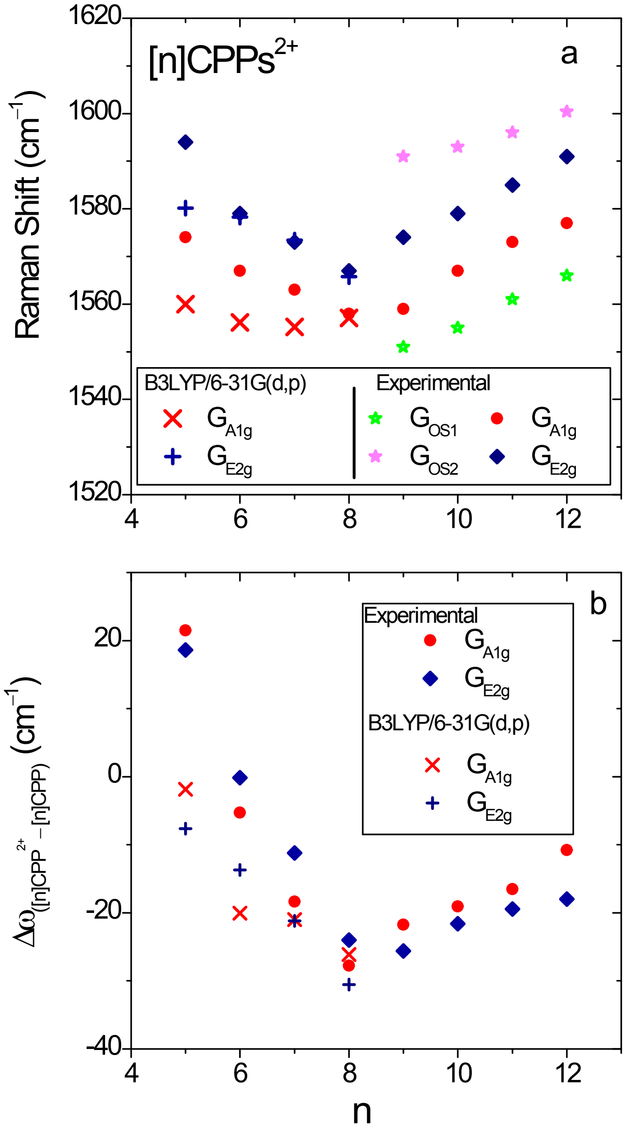

Figure S7. a) V-shape behavior of the Raman shift as a function of *n* of the G bands in the 1520 to 1600 cm^{-1} region for the $[n]CPP^{2+}$ dications. b) Raman shifts of the G_{A1g} and G_{E2g} bands relative to their neutral analogues as a function of *n.* G_{OS1} and G_{OS2} designate side bands which are observed experimentally. These side bands only appear in the open-shell singlet computations and originate from the symmetry breaking discussed in the main text.

Molecular structure of [n]LPP^{2+} dications

Linear oligoparaphenylenes in their oxidized states, cations and dications are known to develop a well-defined quinonoid structure in the π conjugated path, more pronounced for the dications than the mono-cations. In this section we conduct an analysis of the geometries of the dicationic [n]LPPs with *n* = 3 to 6.

[3]LPP^{2+}

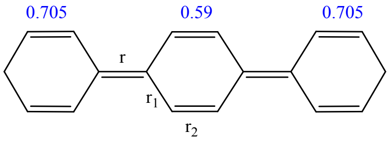

k= 1 2 3

[4]LPP^{2+}

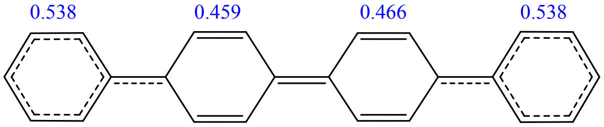

k= 1 2 3 4

[5]LPP^{2+}

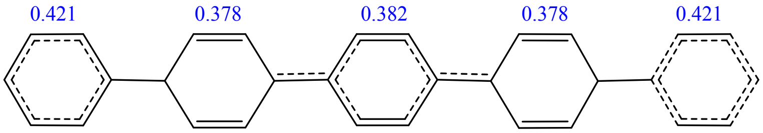

k= 1 2 3 4 5

[6]LPP^{2+}

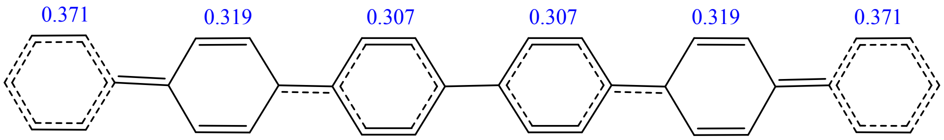

k= 1 2 3 4 5 6

*Figure S8. Valence bond representation of the (U)*B3LYP6-31G(d,p) optimized structure of [3]LPP^{2+} (C_{2h}), [4]LPP^{2+} (C_2), [5]LPP^{2+} (C_{2h}), and [6]LPP^{2+} (D_2), together with the charge distribution on the rings.

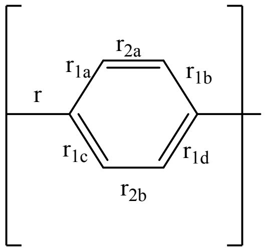

r(CC) interring =r
r_1(CC) ring =(r_{1a}+ r_{1b}+ r_{1c}+ r_{1d})/4
r_2(CC) ring =(r_{2a}+ r_{2b})/2
BLA= r+r_2-2r_1

Figure S9. Scheme of a phenyl unit with the definition of the C-C distances and BLA.

Table S1. Computed parameters of [3]LPP^{2+} (C_{2h}), [4]LPP^{2+} (C_2), [5]LPP$^{•+}$ (C_{2h}), [5]LPP^{2+} (C_{2h}), [6]LPP$^{•+}$ (D_2) and [6]LPP^{2+} (D_2) using *(U)*B3LYP6-31G(d,p). *k* corresponds to the phenyl unit number.

	k	Charge (|e|)	r (Å)	r_1 (Å)	r_2 (Å)	BLA (Å)	θ torsion (°)
**[3]LPP^{2+}**	1	0.705		1.428	1.375	--	9.4
	2	0.590	1.423	1.448	1.361	-0.113	-9.4
	3	0.705	1.423	1.428	1.375	-0.058	--
**[4]LPP^{2+}**	1	0.538	--	1.420	1.380	--	15.6
	2	0.549	1.437	1.438	1.366	-0.073	-9.3
	3	0.466	1.426	1.438	1.366	-0.085	16.5
	4	0.538	1.437	1.420	1.380	-0.022	--
**[5]LPP$^{•+}$**	1	0.192	--	1.403	1.385	--	28.0
	2	0.199	1.468	1.418	1.380	0.013	-21.9
	3	0.217	1.457	1.420	1.378	-0.005	21.9
	4	0.199	1.457	1.417	1.380	0.002	-28.0
	5	0.192	1.468	1.406	1.389	0.045	--
**[5]LPP^{2+}**	1	0.421	--	1.415	1.383	--	-19.3
	2	0.378	1.447	1.431	1.371	-0.045	11.6
	3	0.382	1.435	1.433	1.378	-0.053	-11.6
	4	0.378	1.435	1.431	1.371	-0.056	19.3
	5	0.421	1.447	1.415	1.383	0.000	
**[6]LPP$^{•+}$**	1	0.155	--	1.401	1.394	--	37.0
	2	0.162	1.484	1.405	1.391	0.065	-35.9
	3	0.183	1.482	1.405	1.391	0.063	35.9
	4	0.183	1.482	1.405	1.391	0.062	-35.9
	5	0.162	1.482	1.405	1.391	0.063	37.0
	6	0.155	1.484	1.401	1.394	0.077	
**[6]LPP^{2+}**	1	0.371	--	1.404	1.383	--	19.8
	2	0.319	1.450	1.426	1.374	-0.027	-21.6
	3	0.307	1.456	1.418	1.381	0.001	27.3
	4	0.307	1.466	1.418	1.381	0.011	-21.6
	5	0.319	1.456	1.426	1.374	-0.021	19.8
	6	0.371	1.450	1.404	1.383	0.026	

Raman spectra of [5]- [6]LPP positively charged species

As seen in Figure S10 theoretical simulations for [5]LPP^{2+} and [6]LPP^{2+} predict them as open-shell singlet electronic ground states, as reported in the literature. [5,6] Figures S10a and S11a show the experimental NIR spectra of the neutral, cationic and dicationic forms of [5]LPP and [6]LPP. The IR bands redshift with increasing chain length. The Raman bands in the G band region are shown in Figure S10b and S11b. These Raman bands show a splitting that is associated with the open-shell character of the electronic ground state of both oxidized species.[7]

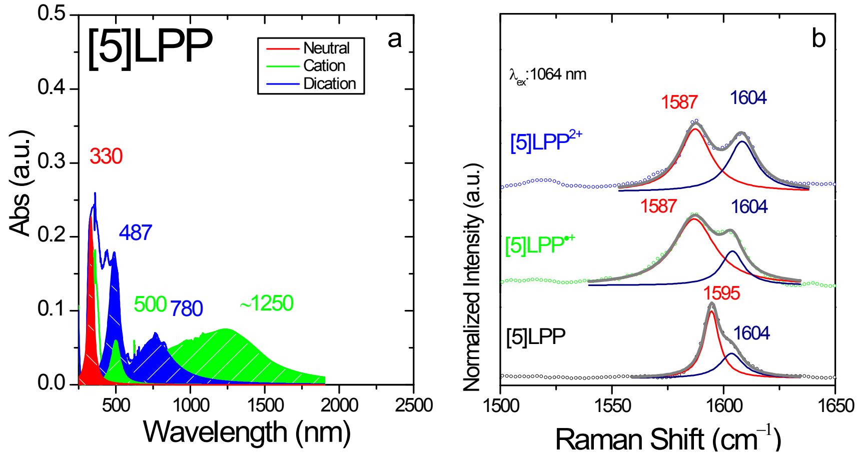

Figure S10. a) [5]LPP in situ UV-Vis-NIR electronic absorption spectra during oxidation. b) [5]LPP in situ Raman spectra during oxidation.

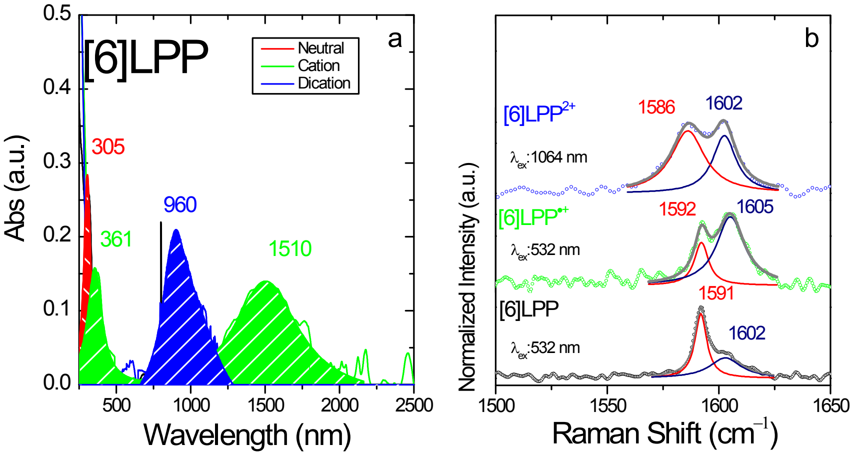

Figure S11. a) [6]LPP in situ UV-Vis-NIR electronic absorption spectra during oxidation. b) [6]LPP in situ Raman spectra during oxidation.

Strain energy of [n]CPP$^{\bullet+}$ and [n]CPP^{2+}: homodesmotic reactions

We have calculated the strain energy of the neutral, radical cations and dications of [n]CPPs by means of the homodesmotic reactions shown in Figures S12. [8,9] The data are summarized in Table S2. This method is the same as used earlier for neutral [n]CPPs by Bachrach et al.[10] and by Segawa et al.[11]

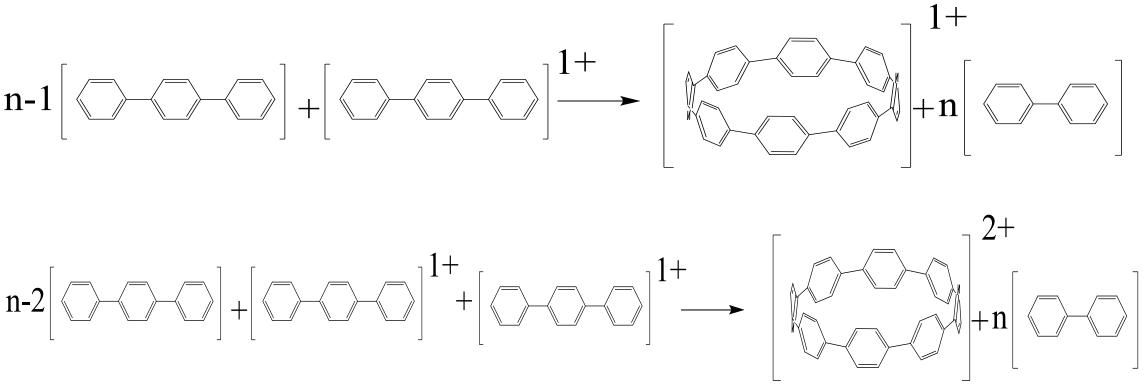

F
igure S12. Homodesmotic reaction for the calculation of strain energies of [*n*]CPP$^{\bullet+}$ and of [*n*]CPP^{2+} dications both at the (U)B3LYP/6-31G(d,p) level.

Table S2. Point groups of symmetry and (U)B3LYP/6-31G(d,p) strain energies of neutral, cationic and dicationic species of [n]CPPs.

n	**[n]CPP**		**[n]CPP$^{\bullet+}$**		**[n]CPP^{2+}**	
	Point group[a]	Strain Energy (kcal/mol)	*Point group*[a]	Strain Energy (kcal/mol)	*Point group*[a]	Strain Energy (kcal/mol)
**20**	C_5~D_{10d}	29.9	C_1~D_{10d}	1.3	C_2	0.3
**18**	C_2~D_{9d}	33.2	C_1~D_{9d}	5.2	C_i	9.5
**16**	C_{4v}~D_{8d}	37.4	C_{4v}~D_{8d}	10.1	C_2	17.8
**15**	C_1~C_2	40.9	C_2	18.8	C_1~C_2	23.2
**14**	D_{7d}	44.8	C_1~C_2	17.6	C_1~C_2	29.1
**13**	C_1	47.2	C_1~C_2	20.5	C_1~C_2	34.6
**12**	D_{6d}	49.9	S_4~D_{6d}	23.9	C_{2v}	40.9
**11**	C_1	55.7	C_1	29.6	C_1	49.1
**10**	D_{5d}	57.9	C_i~C_{2h}	34.4	C_{2h}	57.5
**9**	C_1	70.5	C_1	43.1	C_1	68.5
**8**	D_{4d}	74.6	D_{4d}	49.3	C_{2v}~D_{4d}	79.8
**7**	C_1	86.6	C_1~D_{7h}	59.9	C_1~D_{7h}	94.2
**6**	D_{3d}	98.9	D_{3d}~D_{6h}	72.3	D_{3d}~D_{6h}	111.9
**5**	C_s	119.4	C_s~D_{5h}	89.9	C_s~D_{5h}	135.5
**4**	S_4~D_{2d}	146.9	S_4~D_{4h}	115.8	S_4~D_{4h}	169.3

[a] In the cases where two point groups are indicated, the actual point group corresponds to the first symbol, while the second point group symbol refers to an idealized structure.

Chemical structures and charge distributions on [n]CPP^{2+}

The singlet closed-shell to open-shell transition has a significant impact on the molecular geometries. Hence while the molecular structures for the smaller members of the series are completely uniform and therefore belong to the highest point group of symmetry, [9]CPP^{2+} and larger [n]CPP^{2+} dications have reduced symmetry. This symmetry reduction displays domains with different aromatic-quinonoid characters along the CC bond length alternation paths. Concomitantly with the aromatic-quinonoid differentiation, the positive charge distribution is also not uniform with larger amounts of local charges appearing for the rings with quinonoid character. For the [n]CPP^{2+} dications that have an open-shell singlet ground state configuration, there is a separation of the spin distribution in the macrocycle which is in agreement with diradical character of the dications. Combined with the change in the BLAs these deformed macrocycles develop structures that can be effectively described as two polarons with diradicaloid character or as diradical polaron pairs, DRPP.[12] For $n < 9$, the systems converge toward closed shell ground states with zero spin densities. The main geometrical parameters of their optimized geometries are given in Table S3 for [5]- to [12]CPP^{2+}. In Table S4 we show the orbital topologies of the HOMO orbitals, the positive charge distribution and the electron density distribution for [5]- to [12]CPP^{2+}.

Table S3. Per ring charge distribution, spin density distribution main C-C bond lengths, resulting BLA values (see above) and torsional angles at the (U)B3LYP6-31G(d,p) level :for [5]CPP^{2+} with C_s~D_{5h} , [6]CPP^{2+} with D_{3d}~D_{6h}, [7]CPP^{2+} with C_1~D_{7h}, [8]CPP^{2+} with D_{4d}, [9]CPP^{2+} with C_1, [10]CPP^{2+} with C_{2h}, [11]CPP^{2+} with C_1 and [12]CPP^{2+} with C_{2v}. *k* corresponds to a consecutive numbering of the phenyl. See Figure 9 for C-C distances assignments.

	k	Charge per phenyl unit (|e|)	Spin density	r (Å)	r$_1$ (Å)	r$_2$ (Å)	BLA (Å)	θ torsion (°)
[5]CPP^{2+}	1-5	0.4	--	1.440	1.434	1.371	-0.058	0.0
[6]CPP^{2+}	1-6	0.33	--	1.445	1.430	1.374	-0.040	0.0
[7]CPP^{2+}	1	0.288	--	1.451	1.416	1.384	-0.026	8.8
	2	0.287	--	1.451	1.417	1.383	-0.025	-9.0
	3	0.283	--	1.451	1.418	1.382	-0.024	8.7
	4	0.284	--	1.451	1.417	1.383	-0.025	-7.0
	5	0.284	--	1.451	1.416	1.384	-0.026	2.8
	6	0.283	--	1.451	1.416	1.384	-0.027	2.8
	7	0.287	--	1.451	1.416	1.384	-0.027	-7.0
[8]CPP^{2+}	1-8	0.25	--	1.456	1.423	1.378	-0.012	14.2
[9]CPP^{2+}	1	0.212	0.000	1.463	1.419	1.381	0.007	-19.6
	2	0.226	0.003	1.461	1.421	1.379	-0.002	9.1
	3	0.238	0.004	1.458	1.422	1.378	-0.009	9.3
	4	0.226	0.004	1.458	1.421	1.379	-0.004	-19.1
	5	0.212	0.001	1.461	1.419	1.380	0.004	19.4
	6	0.222	-0.001	1.463	1.419	1.381	0.006	-17.9
	7	0.226	-0.004	1.460	1.421	1.378	-0.004	17.6
	8	0.226	-0.004	1.458	1.421	1.380	-0.003	-17.7
	9	0.218	-0.003	1.460	1.419	1.380	0.002	19.4

	k	Charge per phenyl unit (|e|)	Spin density	r (Å)	r_1 (Å)	r_2 (Å)	BLA (Å)	θ torsion (°)
$[10]CPP^{2+}$	1	0.218	0.200	1.460	1.420	1.379	-0.002	-18.7
	2	0.206	0.160	1.460	1.419	1.380	0.003	21.7
	3	0.185	0.060	1.465	1.416	1.383	0.016	-23.2
	4	0.185	-0.060	1.468	1.416	1.383	0.019	21.3
	5	0.206	-0.160	1.465	1.419	1.380	0.008	-19.0
	6	0.218	-0.200	1.460	1.420	1.379	-0.002	18.7
	7	0.206	-0.160	1.465	1.419	1.380	0.008	-19.0
	8	0.185	-0.060	1.468	1.416	1.383	0.019	21.3
	9	0.185	0.060	1.465	1.416	1.383	0.016	-23.2
	10	0.206	0.160	1.460	1.419	1.380	0.003	21.7
$[11]CPP^{2+}$	1	0.158	-0.030	1.472	1.413	1.385	0.030	24.5
	2	0.177	-0.135	1.470	1.416	1.383	0.020	-22.0
	3	0.201	-0.201	1.463	1.419	1.380	0.006	19.3
	4	0.201	-0.201	1.460	1.419	1.380	0.003	-21.5
	5	0.177	-0.134	1.463	1.416	1.383	0.014	24.9
	6	0.157	-0.030	1.470	1.413	1.385	0.027	-25.1
	7	0.166	0.081	1.472	1.414	1.384	0.027	24.4
	8	0.194	0.178	1.466	1.418	1.381	0.010	-12.6
	9	0.208	0.217	1.461	1.420	1.379	-0.001	-12.3
	10	0.194	0.178	1.461	1.418	1.376	0.001	25.1
	11	0.166	0.080	1.466	1.414	1.384	0.022	-25.0
$[12]CPP^{2+}$	1	0.156	0.099	1.472	1.414	1.384	0.028	-26.6
	2	0.138	0.000	1.472	1.412	1.385	0.034	26.6
	3	0.156	-0.099	1.462	1.414	1.384	0.018	-24.1
	4	0.179	-0.175	1.462	1.417	1.382	0.010	21.2
	5	0.190	-0.200	1.462	1.418	1.380	0.006	-21.2
	6	0.179	-0.175	1.467	1.417	1.382	0.015	24.1
	7	0.156	-0.099	1.472	1.414	1.384	0.028	-26.6
	8	0.138	0.000	1.472	1.412	1.385	0.034	26.6
	9	0.156	0.099	1.462	1.414	1.384	0.018	-24.1
	10	0.179	0.175	1.462	1.417	1.382	0.010	21.2
	11	0.190	0.200	1.462	1.418	1.380	0.006	-21.2
	12	0.179	0.175	1.467	1.417	1.382	0.015	24.1

Table S4. For closed – shell singlet dications B3LYP6-31G(d,p) HOMO and HOMO-1 topologies (these are degenerated), and per ring positive charge distribution. For $n \geq 9$; open-shell singlet dications UB3LYP6-31G(d,p) HOMO topology, spin density distributions (red and blue indicate negative and positive values, respectively) and per ring positive charge distribution

**Closed-shell singlet dications**	**HOMO pattern**	**Charge per phenyl unit (|e|)**
**[5]CPP^{2+} C_s~D_{5h}**	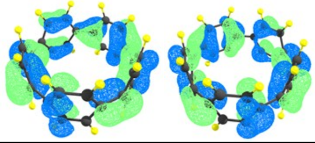	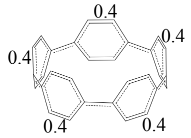
**[6]CPP^{2+} D_{3d}~D_{6h}**	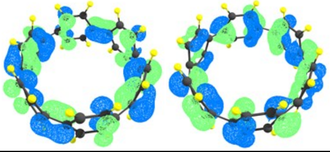	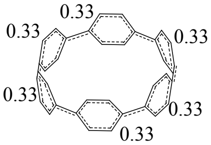
**[7]CPP^{2+} C_1~D_{7h}**	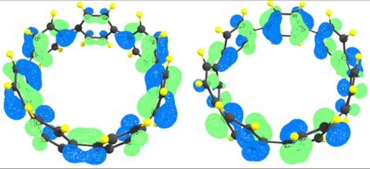	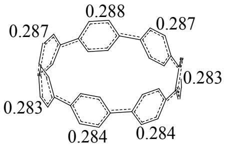
**[8]CPP^{2+} C_{2v}~D_{4d}**	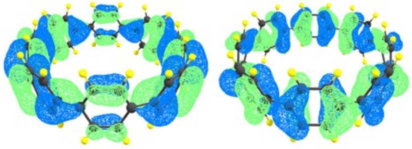	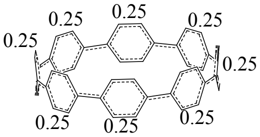

**Open-shell singlet dications**	**HOMO pattern**	**Per ring spin density distributions**	**Charge per phenyl unit (|e|)**
**[9]CPP^{2+} C_1**	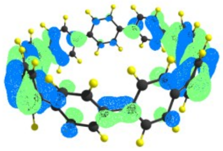	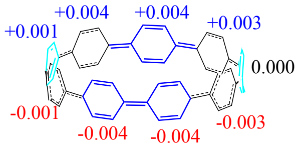	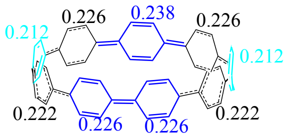
**[10]CPP^{2+} C_{2h}~D_{5h}**	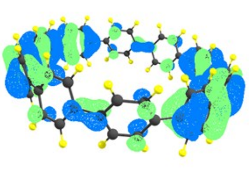	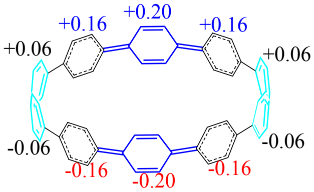	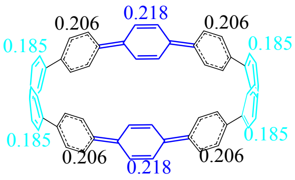
**[11]CPP^{2+} C_1**	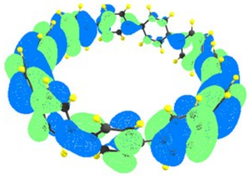	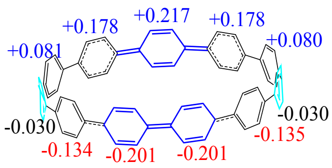	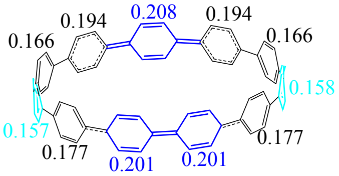
**[12]CPP^{2+} C_{2v}**	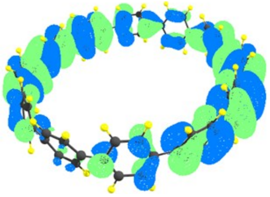	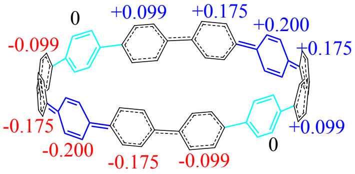	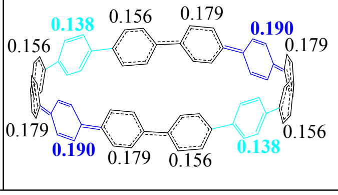

Diameter change from the neutral to the dicationic state

One of the consequences of the symmetry reduction (breaking) for *n*>8 in the [n]CPP^{2+} is the change from a nearly circular shape in the closed-shell molecules (*n*≤8) to a ellipsoid shape for *n*>8. For the latter, we differentiate two main axes, major and minor axis, such as drawn in Figure S13c. In this section we are going to study this distortion. In Figure S13a we display the [n]CPP^{2+} diameters of all species. Similar estimates were obtained by Segawa et al. for neutral [n]CPPs [13].

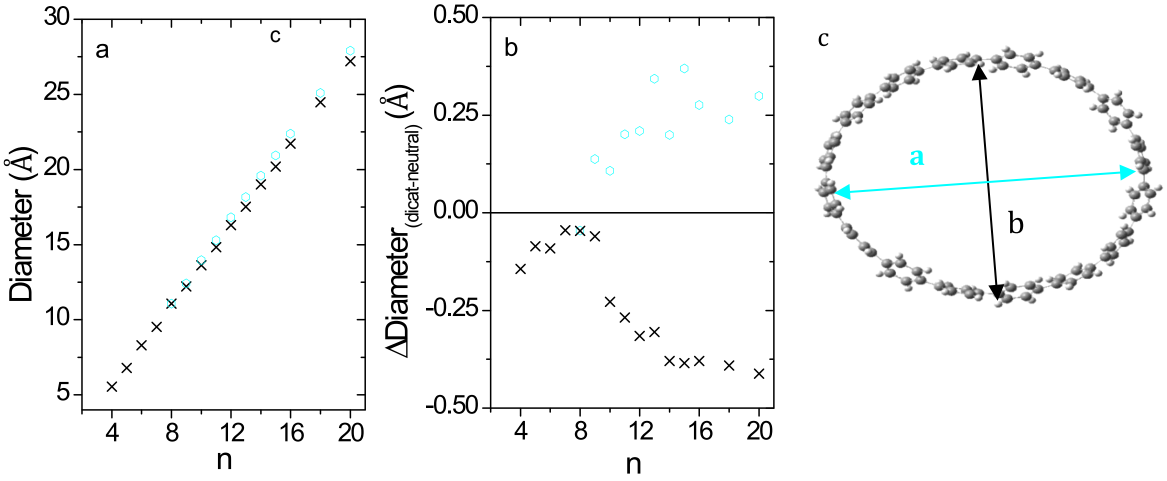

Figure S13. a) Major and minor axis lengths of the [n]CPP^{2+} dications obtained from (U)B3LYP/6-31G(d,p) calculations. Light blue circles (∘) correspond to the major axis (a in Figure S13c) and black crosses (X) correspond to the minor axis (b in Figure S13c). b) Differences between the major and minor axis lengths of the dicationic species compared to the corresponding diameters of the neutral from B3LYP/6-31G(d,p) calculations. Light blue circles (∘) correspond to, ΔD_a=D(neutral)-a, and black crosses (x) correspond to ΔD_b=D(neutral)-b. Since neutrals have a circular shape they are defined with one diameter, D(neutral). c) Scheme indicating the overall ellipsoid structure in [12]CPP^{2+} where the b minor axis has been marked with a black arrow, and the a major axis with a light blue arrow.

Raman spectra of [n]CPP•+ radical cations

Figure S14 shows the Raman data of cationic [n]CPPs in the 1100-1700 cm^{-1} spectral region.

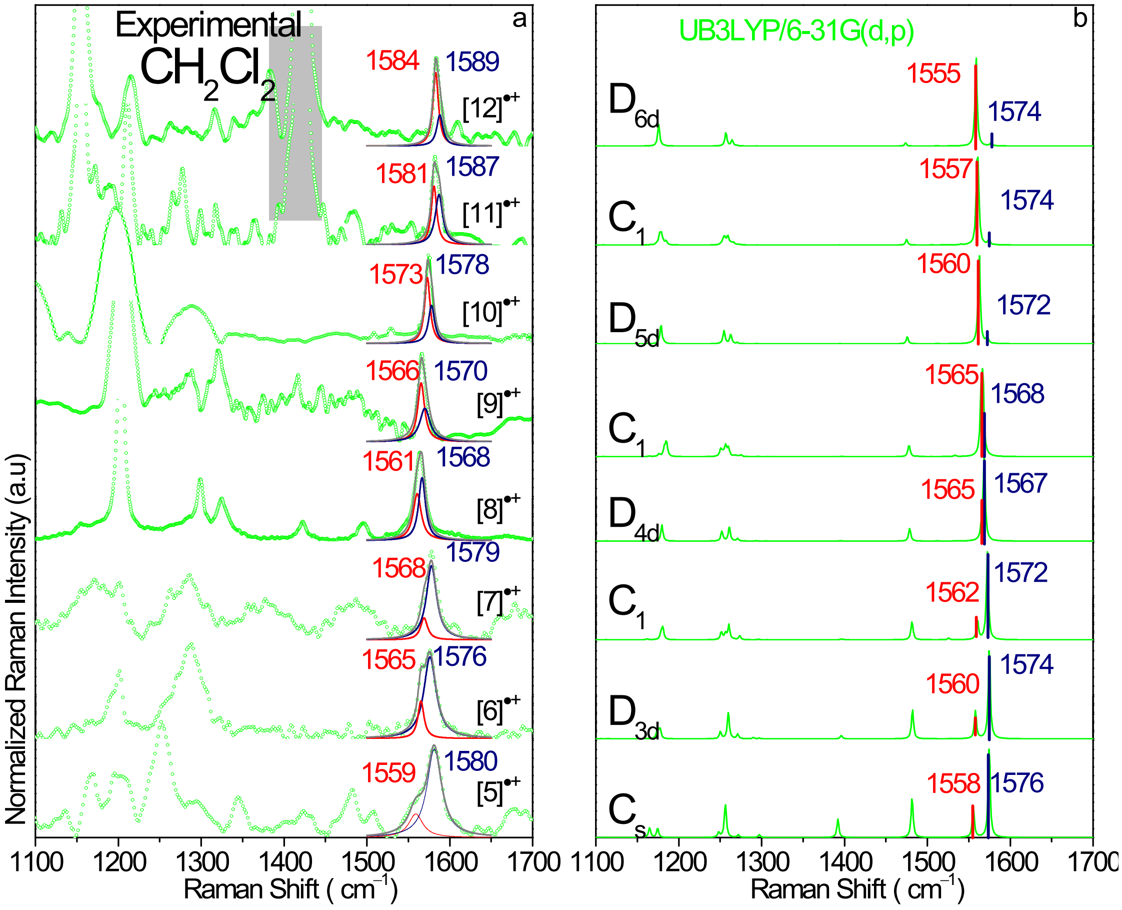

Figure S14. Raman spectra of the cationic species in the 1400-1650 cm^{-1} region, normalized to the G_{A1g} band. a) Experimental and b) computed spectra. Shadowed grey region corresponds to the most intense Raman band of the CH_2Cl_2 solvent which is detected in the near-resonant spectra or when full resonance is not fulfilled.

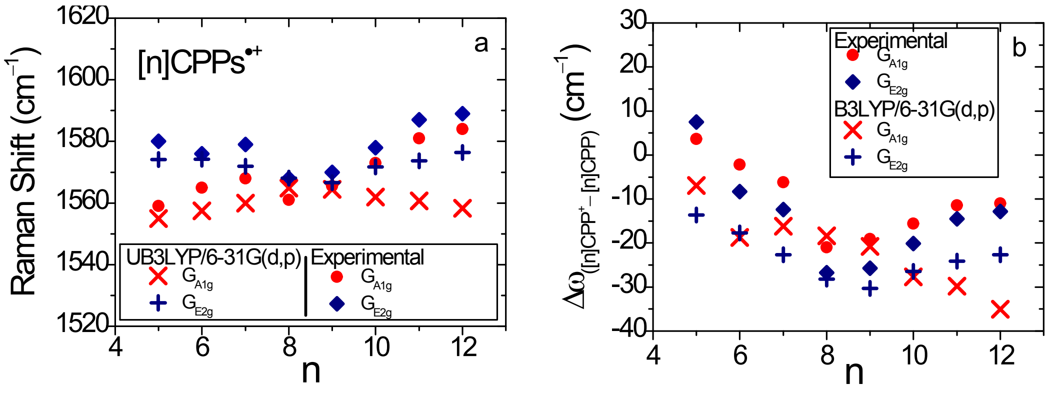

Figure S15. a) Raman shifts for each of the G-like bands as a function of *n*. b) Raman shift changes of the G_{A1g} and G_{E2g} bands relative to the values in the neutral analogues as a function of *n*.

Table S5. *Per ring charge distribution, spin density distribution, main C-C bond lengths, resulting BLA values (see above) and torsional angles at the UB3LYP6-31G(d,p) level for: [5]CPP•+ (C_s~D_{5h}), [6]CPP•+ (D_{3d}~D_{6h}), [7]CPP•+ (C_1~D_{7h}), [8]CPP•+ (D_{4d}), [9]CPP•+ (C_1), [10]CPP•+ (C_{2h}~D_{5h}), [11]CPP•+ $(C)_1$ and for [12]CPP•+ (S_4~D_{6d}).* k *corresponds to the consecutive numbering of phenyl units, see Figure 9 for C-C distances assigments.*

	k	Charge per phenyl unit (|e|)	Spin density	r (Å)	r_1 (Å)	r_2 (Å)	BLA (Å)	θ torsion (°)
[5]CPP•+	1	0.200	0.201	1.467	1.422	1.381	0.004	3.6
	2	0.200	0.200	1.467	1.422	1.381	0.004	5.8
	3	0.200	0.199	1.467	1.422	1.381	0.004	5.7
	4	0.200	0.200	1.467	1.422	1.381	0.004	3.6
	5	0.200	0.201	1.467	1.422	1.381	0.004	0.0
[6]CPP•+	1-6	0.167	0.167	1.468	1.418	1.382	0.014	16.0
[7]CPP•+	1	0.142	0.138	1.471	1.416	1.384	0.022	-19.5
	2	0.147	0.141	1.469	1.417	1.383	0.018	8.8
	3	0.150	0.146	1.469	1.418	1.382	0.016	8.8
	4	0.146	0.148	1.469	1.417	1.383	0.018	-19.5
	5	0.143	0.146	1.469	1.416	1.384	0.021	21.1
	6	0.136	0.141	1.471	1.416	1.384	0.024	-20.0
	7	0.136	0.138	1.471	1.416	1.384	0.024	-7.0
[8]CPP•+	1-8	0.125	0.125	1.471	1.414	1.384	0.027	22.7
[9]CPP•+	1	0.111	0.110	1.472	1.413	1.385	0.031	26.4
	2	0.108	0.105	1.473	1.413	1.385	0.033	-26.6
	3	0.106	0.104	1.474	1.413	1.386	0.034	24.7
	4	0.106	0.104	1.474	1.413	1.386	0.034	-25.9
	5	0.108	0.106	1.471	1.413	1.385	0.031	24.4
	6	0.111	0.111	1.473	1.413	1.385	0.032	-25.5
	7	0.116	0.118	1.472	1.414	1.384	0.028	26.3
	8	0.118	0.125	1.473	1.415	1.384	0.027	-14.1
	9	0.116	0.119	1.473	1.419	1.384	0.019	-13.6
[10]CPP•+	1	0.099	0.099	1.474	1.412	1.386	0.035	-26.3
	2	0.099	0.099	1.474	1.412	1.386	0.035	26.3
	3	0.100	0.100	1.474	1.412	1.386	0.035	-26.3
	4	0.100	0.100	1.474	1.412	1.386	0.035	26.1
	5	0.100	0.100	1.474	1.412	1.386	0.035	-26.1
	6	0.099	0.099	1.474	1.412	1.386	0.035	26.3
	7	0.099	0.099	1.474	1.412	1.386	0.035	-26.3
	8	0.100	0.100	1.474	1.412	1.386	0.035	26.3
	9	0.100	0.100	1.474	1.412	1.386	0.035	26.1
	10	0.100	0.100	1.474	1.412	1.386	0.035	26.1
[11]CPP•+	1	0.088	0.087	1.475	1.411	1.386	0.039	27.5
	2	0.089	0.088	1.475	1.411	1.386	0.039	-26.1
	3	0.092	0.090	1.475	1.411	1.386	0.039	29.9
	4	0.096	0.097	1.474	1.412	1.386	0.035	-17.7
	5	0.097	0.103	1.475	1.413	1.385	0.035	-17.8
	6	0.096	0.097	1.475	1.412	1.386	0.036	30.4
	7	0.092	0.091	1.474	1.411	1.386	0.037	-26.1
	8	0.089	0.088	1.475	1.411	1.386	0.039	27.7
	9	0.088	0.087	1.475	1.411	1.386	0.039	-28.1
	10	0.087	0.086	1.475	1.411	1.386	0.040	26.8
	11	0.087	0.086	1.475	1.411	1.386	0.040	-28.1
[12]CPP•+	1-12	0.083	0.083	1.475	1.411	1.387	0.040	28.1/-28.2

Discussion of the C-C distances

The geometrical and structural deformation of the benzene units for [n]CPPs with n < 9 is significant. The respective computed data are presented in Figure S16.

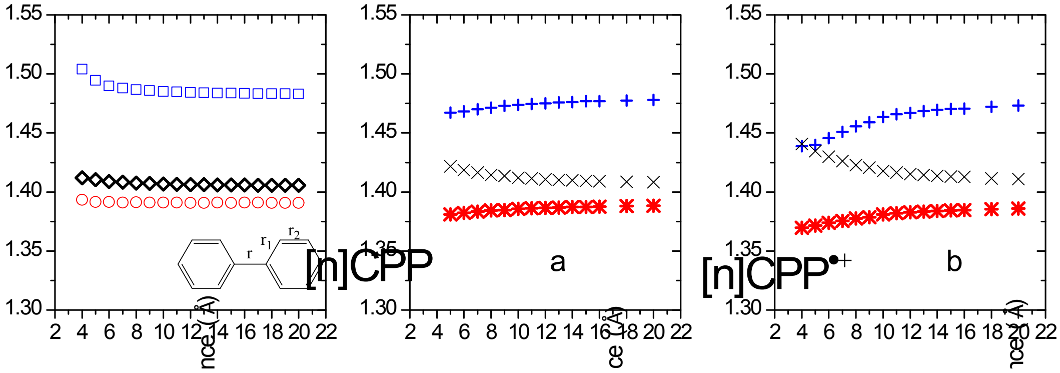

Figure S16. (U)B3LYP6-31G(d,p) average C-C distances (r, r_1, r_2) as a function of *n*. r, r_1 and r_2 are depicted as blue, black and red symbols, respectively. a) Neutral [n]CPP, b) [n]CPP$^{•+}$ cations, and c) [n]CPP^{2+} dications.

NICS values of neutral, cationic and dicationic [n]CPPs.

We used the Nucleus-Independent Chemical Shifts (NICSs) at the (U)B3LYP/6-311+G(2df,p) level to characterize the degree of aromaticity in a given structure. Negative values are typically associated with aromatic systems and positive values for non-aromatic systems.[14] The NICS value of benzene as a reference, $NICS_{benzene}(0)$, is -8 ppm at the ring center while $NICS_{benzene}(1)$ at 1 Å above the ring center is -10 ppm. In Figure S17, we summarize for each [n]CPP in neutral, cationic and dicationic state the obtained NICS at the center of each phenlyl unit and at the center of the [n]CPP cavity, NICS(0) and NICS(C) respectively. NICS(0) reach values as low as that of benzene with decreasing n and increasing charge. However, even though in neutral [n]CPPs the NICS(C) are always non-zero, these rapidly decrease with decreasing n towards –8 ppm for [5]CPP$^{•+}$. For the dications NICS(C) becomes highly negative for n<9.

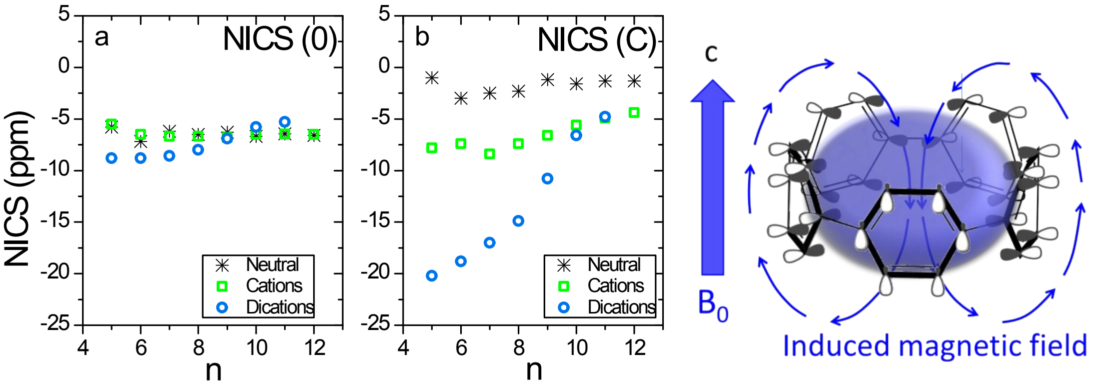

Figure S17. NICS values calculated at the (U)B3LYP/6-311+G(2df,p) theory level vs. *n* for three oxidation states of [n]CPPs. a) NICS(0) average values at the phenyl unit centers. b) NICS(C), at the center of the [n]CPP cavity. c) Scheme representing the induced magnetic field in [5]CPP^{2+}.

Energy calculation of the open shell singlet

Due to the well-known spin contamination problem in spin-unrestricted theory, we provide information on the ⟨S^2⟩ values in Table S6 and the energetics using Yamaguchi's energy correction in Figure S18.

Table S6. Summary of the spin contamination, ⟨S^2⟩ values for each structure.

	**[n]CPPs**				**[n]LPP**	
n	**Neutral ⟨S^2⟩**	**Cation ⟨S^2⟩**	**Dication Open Shell Singlet ⟨S^2⟩**	**Dication Triplet ⟨S^2⟩**	**Dication Open Shell Singlet ⟨S^2⟩**	**Dication Triplet ⟨S^2⟩**
2	--	--	--	--	0.000	2.017
3	--	--	--	--	0.000	2.017
4	--	--	--	--	0.383	2.024
5	0	0.755	0	2.023	0.505	2.021
6	0	0.755	0	2.023	0.845	2.021
7	0	0.754	0	2.021	0.913	2.020
8	0	0.754	0	2.020	0.948	2.019
9	0	0.753	0.276	2.019	0.966	2.017
10	0	0.753	0.524	2.017	0.976	2.015
11	0	0.753	0.681	2.000	0.980	2.014
12	0	0.752	0.742	2.014	0.983	2.013
13	0	0.752	0.802	2.013	0.985	2.011
14	0	0.752	0.833	2.012	0.986	2.013
15	0	0.752	0.851	2.010	0.986	2.009
16	0	0.752	0.858	2.009	0.986	2.009
18	0	0.751	0.877	2.007	0.986	2.007
20	0	0.751	0.891	2.006		2.006

We used the broken spin symmetry method to obtain open-shell singlet states. We estimate the exchange parameter, J, based on the formalisms derived by Noodleman[15] using Yamaguchi modification.[16,17,18]

$$J = -\frac{E(Triplet) - E(\text{Open Shell Singlet})}{\langle S^2 \rangle^{Triplet} - \langle S^2 \rangle^{\text{Open Shell Singlet}}}$$

$$E_{CorrectedOpenShellSinglet} = E(Triplet) + J * \langle S^2 \rangle^{Triplet}$$

E(Triplet) is the energy obtained from the dication optimization with triplet electronic configuration, E(Open Shell Singlet) to the energy obtained from the dication optimization broken spin (UB3LYP). The results are shown in Figure S18. As can be seen, the energy differences obtained with and without the correction are almost identical.

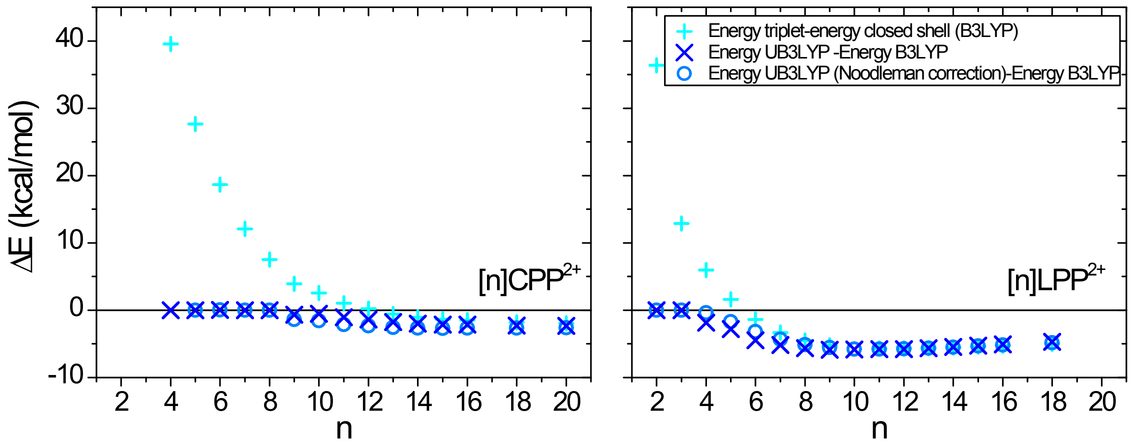

Figure S18. DFT/(U)B3LYP/6-31G(d,p) relative energies (ΔE) between the singlet closed-shell and open-shell); open-shell with the Noodleman correction and with the triplet of: a)[n]CPP^{2+} b))[n]LPP^{2+}. (**X**) correspond to ΔE= E(open-shell singlet dication) – E (closed-shell dication); light blue crosses (+) correspond to ΔE= E(triplet dication) – E (closed shell dication): circles (○) correspond to ΔE= E (corrected open-shell singlet dication) – E (closed-shell dication).

References

[1] T. Iwamoto, Y. Watanabe, Y. Sakamoto, T. Suzuki, S. Yamago, *J. Am. Chem. Soc,*. 2011, **133**, 8354–8361.

[2] P. J. Evans, E. R. Darzi, R. Jasti, *Nature Chem*., 2014, **6**, 404–408.

[3] J. Xia, R. Jasti, *Angew. Chem. Int. Ed*., 2012, **51**, 2474–2476.

[4] Gaussian 09, Revision A.02, M. J. Frisch, G. W. Trucks, H. B. Schlegel, G. E. Scuseria, M. A. Robb, J. R. Cheeseman, G. Scalmani, V. Barone, B. Mennucci, G. A. Petersson, H. Nakatsuji, M. Caricato, X. Li, H. P. Hratchian, A. F. Izmaylov, J. Bloino, G. Zheng, J. L. Sonnenberg, M. Hada, M. Ehara, K. Toyota, R. Fukuda, J. Hasegawa, M. Ishida, T. Nakajima, Y. Honda, O. Kitao, H. Nakai, T. Vreven, J. A. Montgomery, Jr., J. E. Peralta, F. Ogliaro, M. Bearpark, J. J. Heyd, E. Brothers, K. N. Kudin, V. N. Staroverov, R. Kobayashi, J. Normand, K. Raghavachari, A. Rendell, J. C. Burant, S. S. Iyengar, J. Tomasi, M. Cossi, N. Rega, J. M. Millam, M. Klene, J. E. Knox, J. B. Cross, V. Bakken, C. Adamo, J. Jaramillo, R. Gomperts, R. E. Stratmann, O. Yazyev, A. J. Austin, R. Cammi, C. Pomelli, J. W. Ochterski, R. L. Martin, K. Morokuma, V. G. Zakrzewski, G. A. Voth, P. Salvador, J. J. Dannenberg, S. Dapprich, A. D. Daniels, O. Farkas, J. B. Foresman, J. V. Ortiz, J. Cioslowski, and D. J. Fox, Gaussian, Inc., Wallingford CT, 2009.

[5] E. Zojer, J. Cornil, G. Leising, J. L. Brédas, *Phys. Rev. B,* 1999, **59**, 7957–7967.

[6] L. Cuff, C. Cui and M. Kertesz, *J. Am. Chem. Soc*., 1994, **116**, 9269–9274.

[7] M. Peña-Alvarez, P. M. Burrezo, M. Kertesz, T. Iwamoto, S. Yamago, J. Xia, R. Jasti, J. T. López-Navarrete, M. Taravillo, V. G. Baonza and J. Casado, *Angew. Chem. Int. Ed.* ,2014, **53**, 7033–7037.

[8] P. George, M. Trachtman, C. W. Bock, A. M. Brett, *J. Chem. Soc., Perkin Trans. II*, 1976, 1222–1227.

[9] L. Radom, P. C. Hariharan, J. A. Pople, P. V. R. Schleyer, *J. Am. Chem. Soc.* 1976, **98**, 10–14.

[10] S. M. Bachrach and D. Stück, *J. Org. Chem.* ,2010, **75**, 6595–6604.

[11] Y. Segawa, H. Omachi and K. Itami, *Org. Lett*., 2010, **12**, 2262–2265.

[12] J. A. van Haare, E. E. Havinga, J. L. van Dongen, R. A. Janssen, J. Cornil and J. L. Brédas, *Chem. Eur. J.* 1998, **4**, 1509-1522.

[13] Y. Segawa, H. Omachi and K. Itami, *Org. Lett.,* 2010, **12**, 2262-2265.

[14] Z. Chen, C. S. Wannere, C. Corminboeuf, R. Putcha and P. V. R. Schleyer, *Chem. Rev.,* 2005, **105**, 3842-3888.

[15] J. Noodelman, *J. Chem. Phys.,* 1981, **74**, 5734-5743.

[16] K. Yamaguchi, H. Fkui and T. Fueno, *Chem. Lett*. 1986, 625-628.

[17] T. Soda, K. Kitagawa, T. Onishi, Y. Takano, Y. Shigeta, H. Nagao, Y. Yoshioka and K. Yamaguchi, *Chem. Phys. Lett,.* 2000,**319**, 223-230.

[18]M. Podewitz, M. Reiher, Spin interactions in cluster chemistry. Advances in Inorganic Chemistry. Theoretical and Computational Inorganic Chemistry. Edited by R. van Eldik, J. Harvey, 2010, **62**, 177-230.
